# The Impact of CT Reconstruction Parameters on Emphysema Index Quantification, HU‐Based Measurements, and Goddard Score in COPD Assessment: A Prospective Study

**DOI:** 10.1155/ijbi/7436511

**Published:** 2026-01-09

**Authors:** Rahma Saad Mohamed, Ahmed Sayed Abd El Bassset, Ahmed A. G. El-Shahawy

**Affiliations:** ^1^ Department of Radiology & Medical Imaging Technology, Faculty of Applied Health Sciences Technology (AHST), Beni-Suef University, Beni-Suef, Egypt, bsu.edu.eg; ^2^ Department of Radiology, Faculty of Medicine, Beni-Suef University, Beni-Suef, Egypt, bsu.edu.eg; ^3^ Materials Science and Nanotechnology Department, Faculty of Postgraduate Studies for Advanced Sciences (PSAS), Beni-Suef University, Beni-Suef, Egypt, bsu.edu.eg

**Keywords:** chronic obstructive pulmonary disease (COPD), CT imaging, emphysema index (EI), Goddard score (GS), reconstruction parameters, slice thickness (ST)

## Abstract

**Background:**

Quantitative computed tomography (CT) plays a crucial role in assessing emphysema severity in chronic obstructive pulmonary disease (COPD). However, variations in CT reconstruction parameters—slice thickness (ST), kernel selection, field of view (FOV), and reconstruction gaps—can affect emphysema index (EI) quantification, impacting diagnostic accuracy and study comparability.

**Objective:**

This study examines how CT reconstruction parameters influence EI quantification using Hounsfield Unit (HU)‐based measurements and the Goddard Score (GS) to refine imaging protocols for emphysema assessment.

**Methods:**

Low‐dose CT scans were performed on 31 subjects, with images reconstructed using ST (0.6–10 mm), kernel settings (Br and Hr series), FOV ranges (250–370 mm), and reconstruction gaps (0.25–3 mm). EI was defined as the percentage of lung volume with attenuation values below − 950 HU, while GS provided a semi‐quantitative assessment of emphysema severity. Statistical analyses evaluated the effects of reconstruction parameters on EI and GS.

**Results:**

Variations in FOV, kernel selection, and reconstruction gaps had negligible effects on the GS (*p* > 0.05), suggesting that these parameters do not introduce structural distortions in pulmonary imaging. However, ultra‐thin slices (0.6 mm) enhanced the detection of subtle emphysematous changes, slightly increasing GS, though higher image noise may affect interpretation. Additionally, ST significantly influenced EI values due to partial volume effects, with thinner slices yielding lower attenuation values.

**Conclusion:**

These findings confirm the reliability of CT‐based emphysema quantification and highlight the importance of optimizing ST to balance sensitivity and image clarity. Standardized imaging protocols and AI‐driven texture analysis could further enhance quantitative emphysema assessment, improving disease monitoring and therapeutic decision‐making in COPD management.

## 1. Introduction

Chronic obstructive pulmonary disease (COPD) is a progressive lung disorder characterized by persistent airflow limitation and emphysematous tissue destruction. While pulmonary function tests (PFTs) provide valuable functional data, they lack the spatial resolution needed for accurate emphysema assessment, which is particularly crucial for selecting candidates for bronchoscopic lung volume reduction (BLVR). Research indicates that BLVR is most effective in patients with heterogeneous emphysema affecting at least 30% of the target lobe, as sufficient volume reduction improves lung mechanics and gas exchange [1–3].

The EI, derived from quantitative CT, is a validated imaging biomarker for quantifying emphysema severity, monitoring disease progression, and assessing treatment response in COPD [4–6]. It is calculated as the percentage of lung volume with attenuation values below − 950 HU, which reflects alveolar destruction and reduced lung density [7]. However, EI quantification is highly sensitive to CT reconstruction parameters, such as slice thickness, reconstruction algorithms, kernel selection, and radiation dose, which can compromise measurement accuracy and limit cross‐study comparability in both clinical practice and research settings [8–10].

For instance, thinner slice thickness (≤ 1 mm) reduces partial volume effects and improve lesion detection; however, they also increase image noise, potentially leading to overestimated EI values. Likewise, iterative reconstruction (IR) algorithms, while effective in noise reduction, can alter lung density distribution, affecting EI accuracy. Additionally, sharp reconstruction kernels enhance edge definition but may exaggerate low‐attenuation areas, whereas soft kernels reduce noise at the cost of spatial resolution. Radiation dose variations further contribute to measurement discrepancies, as lower‐dose protocols can artificially increase lung density, leading to underestimated emphysema severity. Furthermore, differences in HU thresholds (e.g., − 950 HU vs. − 910 HU) can significantly impact EI calculations, complicating cross‐study comparability [11–13].

In contrast to the HU‐based EI, the GS is a semi‐quantitative visual assessment tool widely used in clinical practice due to its simplicity and accessibility. Introduced by Goddard et al., the GS is particularly valuable for emergency evaluations, preoperative risk stratification, and longitudinal disease monitoring. However, it lacks the precision and granularity of quantitative CT‐based metrics and is prone to inter observer variability, limiting its reliability in accurate emphysema quantification [14].

With the increasing reliance on CT‐based quantitative metrics for COPD management, standardizing reconstruction protocols is essential to enhance reproducibility and ensure accurate emphysema assessment in both clinical practice and multicenter research settings. This study aims to examine the impact of CT reconstruction parameters, specifically kernel selection, slice thickness, FOV, and intervals gaps on EI quantification using HU‐based measurements and the GS. A comprehensive understanding of these effects is crucial for optimizing imaging methodologies and enhancing the reliability of CT‐based emphysema quantification.

## 2. Materials and Methods

### 2.1. Subjects

A low‐radiation‐dose multi‐detector computed tomography (MDCT) scan was performed on 31 subjects (28 males, 3 females) to assess the severity of pulmonary emphysema. Participants exhibited varying degrees of emphysema, ranging from mild to severe. The inclusion of patients with different severities enabled a more comprehensive evaluation of disease progression. However, restricting the analysis to severe cases could introduce bias and limit the generalizability of the findings to individuals with milder forms of the disease. To minimize potential confounding factors, individuals with a history of asthma, diffuse lung diseases other than emphysema, or clinical signs of an active lung infection were excluded. Patients were recruited from EL‐Assema Scan Center in Beni‐Suef, Egypt, between April and October 2024. The study was approved by the local ethics committee, approval number (FMBSUREC/01102024).

### 2.2. CT Scan Protocol and Image Reconstruction Parameters

CT scans were performed using a multi‐detector scanner (Siemens Somatom Go. Now 16, Medical Solutions, Erlangen, Germany) that underwent daily calibration checks. The scanner’s detector array comprises 16 elements, each measuring 0.7 mm in width. The scans were acquired at full inspiration without intravenous contrast, using a low‐radiation‐dose technique with the following parameters: tube voltage, 130 kVp; effective tube current, 30 mAs; tube current‐time product, 43 mAs; and pitch factor, 1.

#### 2.2.1. Image Reconstruction Details

CT axial images were reconstructed using various slice thickness, reconstruction kernels, FOV settings, and reconstruction gaps, with all other imaging parameters kept constant: slice thickness: 0.6 mm, 0.8 mm, 1 mm, 1.5 mm, 2 mm, 3 mm, 4 mm, 5 mm, 6 mm, 7 mm, 8 mm, and 10 mm. Reconstruction kernels: Br (body regular) series: Br44, Br48, Br56, Br60, Br64; Hr (high‐resolution) series: Hr36, Hr44, Hr56, Hr64. FOV settings: ranged from 250 to 370 mm in 10 mm increments. Reconstruction gaps: applied in 0.25 mm increments, ranging from 0.25 to 3 mm. All other imaging parameters remained constant across reconstructions.

#### 2.2.2. EI Quantification HU‐Based Measurements’

For emphysema quantification, the CT emphysema index (EI) was defined as the proportion of lung pixels with attenuation values below − 950 HU. This threshold is widely recognized in quantitative CT (qCT) analysis as a surrogate marker of alveolar destruction and reduced lung density. Compared to visual scoring, the CT EI provides a more objective and reproducible measure of lung tissue loss, enhancing the accuracy of disease progression assessment.

To measure the attenuation coefficient values of lung pixels in each patient, a distinct region of interest (ROI) was symmetrically placed over the emphysema area using the lung window, as illustrated in (Supplementary Figure 1). For each ROI, the mean value was recorded. Since 31 patients were included in the study, a total of 31 means were obtained for each investigated parameter. The median of these values are reported in the tables. This approach improves the accuracy of attenuation coefficient quantification by minimizing boundary‐related signal inconsistencies.

### 2.3. Assessing the Robustness of the GS Across CT Imaging Conditions

To evaluate the consistency of the GS across different CT acquisition and reconstruction settings, a systematic analysis was conducted. The following parameters were varied: slice thickness, reconstruction kernels (soft tissue vs. high‐resolution), FOV (250 to 370 mm in 10 mm increments), and reconstruction gaps (0.25 mm to 3 mm in 0.25 mm increments). Given that variations in CT acquisition parameters can significantly impact emphysema quantification, this study systematically evaluates these factors to determine whether the GS remains a robust and reliable metric across diverse imaging conditions.

Beyond quantitative assessment, optimizing reconstruction parameters enhances the accurate detection of low‐attenuation areas while minimizing artifacts that could interfere with emphysema grading. From a clinical standpoint, ensuring the reproducibility of the GS across different scanners and imaging protocols strengthens its applicability in multi‐center studies and real‐world clinical practice.

#### 2.3.1. CT Image Analysis and EI Quantification–Based GS Assessment

In the current study, we applied GS for emphysema assessment. CT images were analyzed using Lung Analysis Software (Synapse 3D Workstation). The tool offers an automated and standardized approach for lung segmentation axial, sagittal, and coronal (Supplementary Figure 2), labelling the low attenuation area (LAA) with − 950 HU axial, sagittal, and coronal (Supplementary Figure 3), 3D Volume Rendering quantification (Supplementary Figure 4), 3D Volume Rendering clustering the LAA‐based volume (Supplementary Figure 5), and histogram‐based attenuation analysis (Supplementary Figure 6), enabling precise detection of structural changes in lung parenchyma.

Automation minimizes observer bias, enhances quantitative assessment, and improves the accuracy and reproducibility of emphysema evaluation.

To achieve a precise assessment of regional emphysema distribution, the lung fields were divided into three sections per lung: the upper section (corresponding to the level of the aortic arch), the middle section (at the level of the carina), and the lower section (aligned with the upper diaphragm), as depicted in Supplementary Figure 7. Each section was assigned a score based on the degree of emphysematous involvement. The percentage of low‐attenuation areas within the upper, middle, and lower zones of both lungs was calculated (Supplementary Figure 3), and the corresponding scores were summed to determine the final GS. The grading criteria were as follows: 0%–5% (score 0, normal); 5%–25% (score 1, mild emphysema, total score range 1–7); 25%–50% (score 2, moderate emphysema, total score range 8–15); 50%–75% (score 3, severe emphysema, total score range 8–15); and 75%–100% (score 4, very severe emphysema) [15].

## 3. Results

### 3.1. Impact of Reconstructed Parameters on EI Quantification HU‐Based Measurements’

#### 3.1.1. Slice Thickness

Table 1 presents the median HU values and interquartile ranges (IQRs) for CT slice thickness ranging from 0.60 to 10.00 mm. The results demonstrate a consistent increase in median HU values with increasing slice thickness. The thinnest slice (0.60 mm) exhibited the lowest median HU (− 996.0 HU, IQR − 1005.0 to − 981.5), while the thickest slice (10.00 mm) recorded the highest (− 959.0 HU, IQR − 990.0 to − 890.5). One‐way ANOVA revealed statistically significant differences (*p* < 0.05) between the 0.60 mm slice and all thicker slices (5.00–10.00 mm), as well as between the 10.00 mm slice and all thinner slices. This upward shift in HU values with increasing slice thickness is attributed to partial volume effects, wherein signals from adjacent tissues are averaged, leading to an overestimation of tissue density and a reduction in spatial resolution. These findings emphasize the critical need for standardizing CT slice thickness to ensure accurate and reproducible HU‐based measurements, particularly in the quantitative evaluation of lung density and emphysema severity.

**Table 1:** Statistical comparison of Hounsfield unit (HU) values across different slice thicknesses, highlighting significant differences (*p* < 0.05).

**Slice thickness**	**Median HU (IQR)**
0.60 mm	− 996.0 (− 1005.0, − 981.50)
0.80 mm	− 991.5 (− 997.0, − 975.75)
1.00 mm	− 990.5000 (− 999.25, − 977.75)
1.50 mm	− 991.0 (− 997.0, − 983.25)
2.00 mm	− 987.5 (− 994.0, − 978.25)
3.00 mm	− 986.0 (− 995.5, − 967.25)
4.00 mm	− 986.5 (− 994.5, − 969.5)
5.00 mm	− 985.0 (− 993.0, − 957.25)
6.00 mm	− 978.0 (− 992.25, − 951.75)
7.00 mm	− 972.0 (− 990.25, − 938.0)
8.00 mm	− 964.50 (− 990.0, − 918.75)
10.00 mm	− 959.0 (− 990.0, − 890.5)

**Figure 1:**
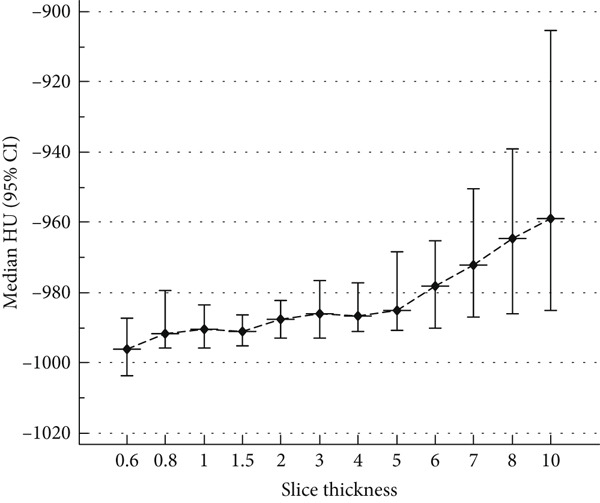
Relationship between CT slice thickness and median HU values. The data demonstrate a progressive increase in median HU values as slice thickness increases from 0.6 mm to 10.0 mm, thinner slices (0.6 mm to 2.0 mm) exhibit relatively stable HU values with narrow confidence intervals, while slices thicker than 3.0 mm show an upward shift in HU values due to partial volume effects.

Figure 1 illustrates the relationship between CT slice thickness and median HU values, with 95% confidence intervals (CIs) represented as error bars. The data demonstrate a progressive increase in median HU values as slice thickness increases from 0.60 to 10.00 mm. Thin slices (e.g., 0.60–2.00 mm) exhibit relatively stable HU values with narrow confidence intervals, reflecting higher measurement precision and lower variability. In contrast, slices thicker than 3.00 mm show a noticeable upward shift in HU values, accompanied by wider confidence intervals, indicating increased variability and potential partial volume effects. The pronounced rise in HU values beyond 5.00 mm underscores the impact of reduced spatial resolution on tissue density quantification. These findings highlight the critical importance of selecting an appropriate slice thickness to ensure accurate and reproducible HU‐based measurements, particularly in quantitative assessments of lung density and emphysema severity.

Table 2 presents the correlation analysis between CT slice thickness and median HU values, which demonstrates a moderate positive correlation, with a correlation coefficient of 0.405. This relationship indicates that as slice thickness increases, HU values systematically rise. The highly significant *p* value (< 0.001) confirms the robustness of this association and rules out the possibility of random variation. This finding aligns with the well‐established partial volume effect, where thicker slices result in signal averaging from adjacent tissues, leading to an artificial elevation of HU values and a reduction in spatial resolution. These results emphasize the critical need to standardize CT slice thickness to ensure accurate and reproducible HU measurements, particularly in quantitative imaging studies, such as lung density evaluation and emphysema severity assessment, where even minor variations in HU values can affect clinical decision‐making.

**Table 2:** Correlation analysis between Hounsfield unit (HU) values and slice thickness.

		**HU**
Slice thickness	Correlation coefficient	0.405 ^∗∗^
	*p* value	< 0.001

^∗∗^indicates a statistical significance level of *p* < 0.001.

**Figure 2:**
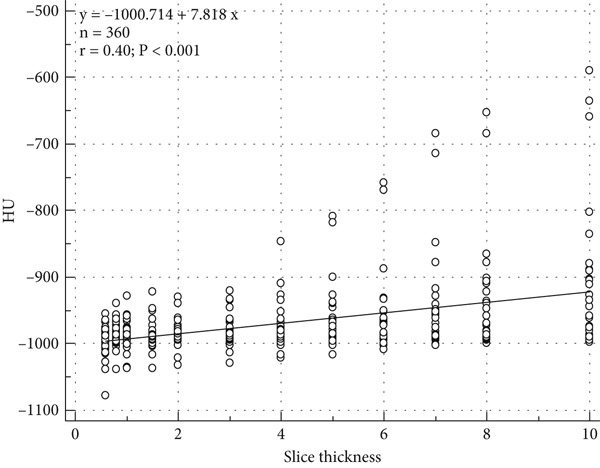
Scatter plot illustrating the positive correlation between CT slice thickness and median HU values (*r* = 0.40, *p* < 0.001). The regression model (*y* = −1000.714 + 7.818*x*) suggests that HU values increase by approximately 7.8 units per millimeter of slice thickness This trend is primarily attributed to partial volume averaging, where adjacent tissue signals blend within thicker slices, leading to elevated HU values and reduced spatial resolution.

Figure 2 demonstrates the positive correlation between CT slice thickness and median HU values (*r* = 0.40, *p* < 0.001). The linear regression model (*y* = −1000.714 + 7.818*x*) indicates that HU values increase by approximately 7.8 units per millimeter of slice thickness. This upward trend is primarily attributed to partial volume averaging, where signals from adjacent tissues are blended within thicker slices, leading to artificially elevated HU values and diminished spatial resolution. Notably, thinner slices (0.60–2.00 mm) exhibit lower variability and narrower confidence intervals, reflecting higher measurement precision. In contrast, thicker slices (≥ 5.00 mm) show greater dispersion and increased variability, which can compromise the accuracy of tissue density quantification. These findings emphasize the necessity of standardizing CT slice thickness to enhance the reproducibility and reliability of HU‐based measurements, particularly in quantitative imaging studies, such as lung density analysis and emphysema severity assessment, where subtle changes in HU values are clinically significant. Supplementary Figure 8 displays the HU‐based measurements at different reconstructed slice thickness using lung window.

#### 3.1.2. Kernels

Table 3 presents the correlation between CT reconstruction kernels and median HU values, expressed as IQR. The results demonstrate a significant reduction in HU values with HR kernels (Hr 36, − 1045.5 HU; Hr 44, − 1042.5 HU) compared to Br kernels (Br 44, − 998.0 HU; Br 64, − 990.0 HU) (ANOVA, *p* < 0.001; post hoc Tukey test, *p* < 0.05). This reduction is primarily attributed to the enhanced edge detection and noise suppression algorithms inherent in HR kernels, which improve the visualization of low‐density structures by minimizing the partial volume effect. Notably, the Br 44 kernel exhibits a statistically significant deviation from other Br kernels, likely due to variations in its smoothing filter and noise‐handling mechanisms. These findings underscore the profound impact of kernel selection on HU accuracy, emphasizing the importance of standardization in quantitative CT imaging, particularly for lung density evaluation and emphysema quantification, where subtle HU variations can critically influence diagnostic accuracy and disease progression monitoring.

**Table 3:** Comparison of Hounsfield unit (HU) values across different reconstruction kernels, highlighting statistically significant differences (*p* < 0.05).

**Kernel**	**Median HU (IQR)**
Br 44	− 998.0 (− 1008.5, − 986.5)
Br 48	− 988.0 (− 994.0, − 979.5)
Br 56	− 987.0 (− 994.2, − 977.75)
Br 60	− 988.5 (− 996.5, − 968.75)
Br 64	− 990.0 (− 998.25, − 979.0)
Hr 36	− 1045.5 (− 1083.75, − 1005.0)
Hr 44	− 1042.5 (− 1071.0, − 1001.5)
Hr 56	− 987.5 (− 991.25, − 976.75)
Hr 64	− 989.0 (− 999.0, − 977.5)

**Figure 3:**
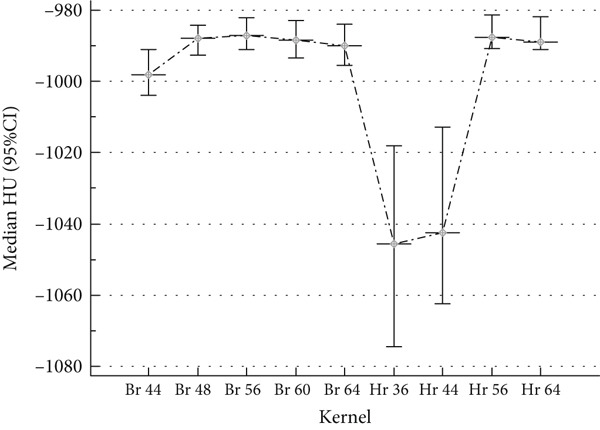
Relationship between CT reconstruction kernel type and median HU values, with 95% confidence intervals. High‐resolution (Hr) kernels exhibit higher median HU values compared to standard (Br) kernels, with increased variability due to noise amplification and enhanced edge definition.

Figure 3 illustrates the relationship between CT reconstruction kernel type and median HU values, with 95% CI represented by error bars. The results demonstrate a systematic variation in HU values across different reconstruction kernels, highlighting the influence of kernel selection on quantitative CT measurements. High‐resolution kernels exhibit lower median HU values compared to standard kernels, with a pronounced increase in variability, as indicated by the wider confidence intervals. This variability likely stems from noise amplification and enhanced edge definition, characteristic of HR kernels, which can lead to overestimation of tissue density. Notably, the substantial divergence observed for specific kernel settings (HR 36, HR 44) underscores the potential impact of reconstruction parameters on clinical and research applications, particularly in lung density quantification and emphysema assessment. The marked fluctuations in HU values for certain HR kernels suggest the need for careful kernel selection to ensure measurement consistency and diagnostic accuracy. These findings emphasize the necessity of standardizing reconstruction protocols to minimize inter‐scanner variability and enhance the reliability of quantitative CT assessments. These findings, further illustrated in Supplementary Figure 9, highlight the substantial impact of kernel choice on HU accuracy and variability, reinforcing the need to standardize reconstruction protocols for consistent and reliable emphysema quantification and lung density assessment across clinical and research settings.

#### 3.1.3. FOV

Table 4 presents the median HU values and IQR across different FOV settings, ranging from 250 to 370 mm. The analysis reveals no statistically significant difference in median HU values across the various FOV settings (*p* = 0.999), indicating that FOV adjustments do not influence HU‐based measurements. The median HU values remained stable, ranging from − 989.5 HU to − 992.0 HU, with minimal variability within the IQRs and substantial overlap between groups. This consistency can be attributed to the homogeneous distribution of attenuation values within the scanned region, which mitigates the impact of FOV size on HU quantification. These findings are particularly significant in quantitative CT imaging, as they confirm that expanding the FOV does not compromise the accuracy or reproducibility of HU‐based measurements. This is especially crucial in lung density evaluation and emphysema quantification, where subtle HU fluctuations can affect diagnostic accuracy and disease progression monitoring. Moreover, the observed stability across different FOV settings enhances the reliability of CT‐based biomarkers in multi‐center studies, where variability in scanner protocols is often unavoidable.

**Table 4:** Comparison of Hounsfield unit (HU) values across different fields of view (FOV), indicating statistically non‐significant differences (*p* = 0.999).

**FOV**	**Median HU (IQR)**
250.00	− 991.5(− 995.0, − 970.0)
260.00	− 989.50(− 995.25, − 967.0)
270.00	− 991.50(− 995.25, − 977.25)
280.00	− 991.0(− 997.0, − 977.5)
290.00	− 991.5(− 995.0, − 973.0)
300.00	− 990.0(− 995.25, − 973.75)
310.00	− 992.0(− 997.0, − 978.75)
320.00	− 991.5(− 996.0, − 979.25)
330.00	− 989.5(− 995.25, − 970.5)
340.00	− 991.0(− 996.0, − 972.75)
350.00	− 990.0(− 996.0, − 976.25)
360.00	− 991.5(− 996.25, − 978.0)
370.00	− 989.5(− 995.25, − 969.75)

**Figure 4:**
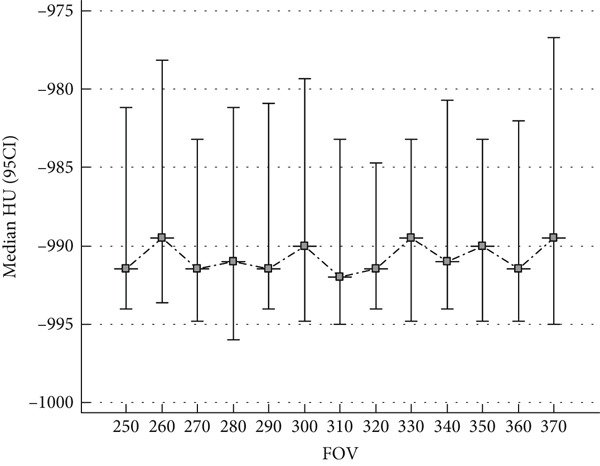
Relationship between FOV and median HU values, with 95% confidence intervals. The data demonstrate relatively stable median HU values across varying FOV settings, indicating minimal impact of FOV on emphysema quantification. However, increased confidence intervals at larger FOVs suggest increased variability, likely due to partial volume effects.

Figure 4 illustrates the relation between FOV and median HU values, with 95% CI depicted as error bars. The results demonstrate relatively stable median HU values across varying FOV settings. However, the notable expansion of confidence intervals at larger FOVs indicates increased variability, which can be attributed to the partial volume effect and signal averaging. This phenomenon arises as larger FOVs capture a more heterogeneous anatomical region, leading to reduced accuracy and precision in HU‐based measurements. These findings emphasize the critical importance of optimizing FOV settings to mitigate variability and enhance the reliability of quantitative HU analysis, particularly in CT‐based lung density assessment and emphysema evaluation.

Table 5 reports the correlation between HU and FOV, revealing a correlation coefficient of − 0.00001 and a *p* value of 0.936. The correlation coefficient, which is nearly zero, indicates a negligible association between HU values and FOV. Furthermore, the high *p* value suggests that this correlation is not statistically significant, implying that any fluctuations in HU‐based measurements across varying FOV settings are likely due to random variation rather than a true underlying effect. These findings are of particular importance in radiological imaging, as they confirm the robustness and stability of HU‐based measurements, irrespective of FOV adjustments. This consistency is critical for enhancing the reliability and reproducibility of quantitative imaging analyses, thereby supporting the standardization of imaging protocols in both clinical practice and research environments.

**Table 5:** Correlation analysis between Hounsfield unit (HU) values and field of view (FOV), indicating a non‐significant correlation (*p* = 0.936).

		**HU**
FOV	Correlation coefficient	− 0.00001
	*p* value	0.936

**Figure 5:**
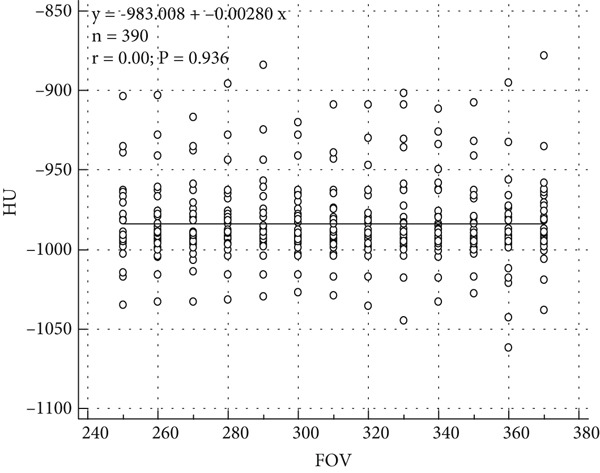
Scatter plot illustrating the correlation between FOV and median HU values. The correlation coefficient (*r* = 0.00, *p* = 0.936) indicates no significant association between FOV and HU values, confirming the stability of HU‐based measurements across different FOV settings.

Figure 5 is the scatter plot illustrating the relationship between HU and FOV, with a regression equation. The correlation coefficient (*r* = 0.00) indicates a complete lack of association between HU and FOV, while the high *p* value of 0.936 confirms that this correlation is not statistically significant. The scattered distribution of data points further supports the conclusion that changes in FOV have no measurable impact on HU values. These findings are crucial in radiological imaging, as they demonstrate the stability and reliability of HU‐based measurements across varying FOV settings. Such consistency is essential for enhancing the accuracy and reproducibility of quantitative imaging analyses, thereby supporting the standardization of imaging protocols in both clinical practice and research environments. Supplementary Figure 10 displays the HU‐based measurements at different reconstructed FOVs using lung window.

#### 3.1.4. Gap

Table 6 presents the median HU and IQR across different gap sizes, ranging from 0.25 to 3 mm. The data reveal a gradual increase in median HU values as the gap size widens, from − 997.25 HU at 0.25 mm to − 994.25 HU at 3 mm. Despite this upward trend, the *p* value of 0.966 indicates that the differences are not statistically significant, suggesting that the variations in HU values are likely due to random fluctuations rather than a systematic effect of gap size. Moreover, the IQR remains relatively stable across the different gap sizes, reflecting consistent data distribution. These findings imply that altering the gap size within this range does not substantially influence HU‐based measurements. However, future studies with larger sample sizes and more controlled conditions may be necessary to confirm these observations and identify potential underlying factors affecting HU values.

**Table 6:** Comparison of Hounsfield unit (HU) values across different gap sizes, indicating statistically non‐significant differences (*p* = 0.966).

**Gap**	**Median HU (IQR)**
0.25 mm	− 997.25 (− 990.0, − 982.0)
0.5 mm	− 998.5 (− 989.5, − 981.5)
0.75 mm	− 998.25 (− 990.0, − 976.5)
1 mm	− 996.0 (− 989.0, − 969.0)
1.25 mm	− 996.25 (− 989.5, − 977.75)
1.5 mm	− 995.25 (− 989.0, − 973.5)
1.75 mm	− 994.5 (− 987.0, − 970.5)
2 mm	− 996.5 (− 989.5, − 971.25)
2.25 mm	− 995.5 (− 989.0, − 979.0)
2.5 mm	− 996.25 (− 987.5, − 977.5)
2.75 mm	− 995.0 (− 988.0, − 975.75)
3 mm	− 994.25(− 985.5, − 969.0)

Figure 6a is the line plot illustrating the median HU values across varying gap sizes, with 95% CI. The data exhibit minor fluctuations in HU values as the gap size increases from 0.25 to 3 mm; however, no clear trend or significant deviation is observed. The relatively wide confidence intervals reflect substantial variability in HU‐based measurements, potentially arising from factors such as image noise or variations in material density. The considerable overlap between confidence intervals further suggests that the differences in HU values across gap sizes are statistically insignificant. These findings highlight the stability and robustness of HU‐based measurements, regardless of gap size variation. This consistency is crucial for enhancing the accuracy and reliability of quantitative imaging analyses, particularly in clinical diagnostics and research applications where precise HU values are essential for standardization and reproducibility.

Figure 6(a) Line plot illustrating the median HU values across varying reconstruction gap sizes, with 95% confidence intervals. The data show minor fluctuations in HU values as the gap size increases from 0.25 to 3 mm, but no clear trend or significant deviation is observed, suggesting stability in HU‐based measurements. (b) Scatter plot illustrating the relationship between HU values and reconstruction gap size. The linear regression equation suggests a weak positive trend (*r* = 0.11, *p* = 0.125), but the correlation is not statistically significant, indicating that gap size has minimal impact on HU quantification.

**Figure:**
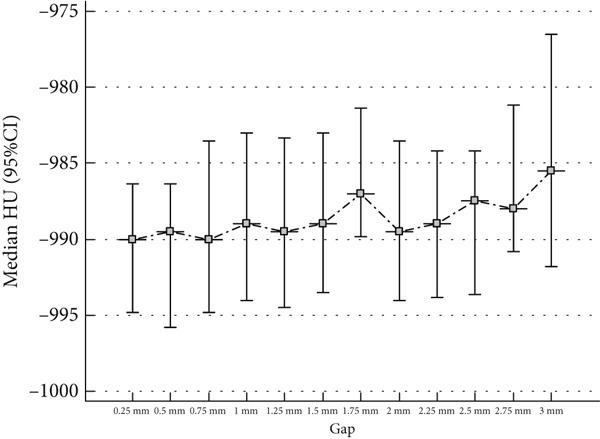
(a)

**Figure:**
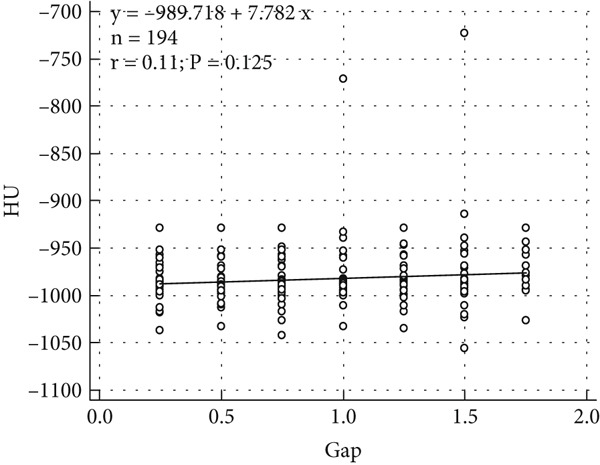
(b)

Table 7 presents the correlation between HU and gap size, with a correlation coefficient of 0.111 and a *p* value of 0.125. The weak positive correlation coefficient indicates a negligible association between HU values and gap size. However, the lack of statistical significance, as evidenced by the *p* value exceeding the conventional threshold of 0.05, suggests that this relationship is not meaningful and is likely due to random variability. These findings demonstrate the robustness and stability of HU‐based measurements across varying gap sizes, highlighting the reliability of the imaging process. Such stability is fundamental for ensuring the accuracy and reproducibility of quantitative imaging analyses and plays a critical role in standardizing imaging protocols for both clinical diagnostics and research applications.

**Table 7:** Correlation between HU and gap: indicating a non‐significant correlation (*p* = 0.125).

		**HU**
Gap	Correlation coefficient	0.111
	*p* value	0.125

Figure 6b is the scatter plot demonstrating the relationship between HU and gap size. The linear regression equation indicates a weak positive trend, as reflected in the correlation coefficient (*r* = 0.11). However, the *p* value of 0.125 exceeds the standard significance threshold of 0.05, suggesting that this correlation is not statistically significant. The wide dispersion of data points around the regression line further supports this finding. These results imply that variations in gap size have a limited effect on HU values. However, given the weak correlation, future studies with larger sample sizes and controlled variables are necessary to confirm these observations. This stability in HU‐based measurements is essential for ensuring accuracy and consistency in quantitative imaging analyses. Supplementary Figure 11 displays the HU‐based measurements at different reconstructed gaps using lung window.

### 3.2. Impact of Reconstructed Parameters on EI Quantification–Based GS Measurements

#### 3.2.1. Case Study1

In one of the 31 cases analyzed in the present study, as shown in Table 8, variations in slice thickness had minimal impact on lung volume and HU values. However, LAA% exhibited slight fluctuations, with a mild GS detected only at 0.6 mm. Changes in the FOV did not significantly affect HU values or LAA%, which remained at 0.6% when lung volume data were available. The GS remained consistently normal, though some volume data were missing. Increasing kernel sharpness correlated with a rise in LAA%, reaching 3.1% at Br 68, while lung volume showed slight reductions. HU values remained stable, and the GS was normal across all kernel settings. Lung volume and HU values were consistent across different gap sizes, with minor LAA% increases at 2.75 mm and 3 mm. The GS remained normal, indicating no structural lung abnormalities. All recorded measurements were included in a supplementary file.

**Table 8:** Influence of slice thickness, FOV, reconstruction kernel, and gap size on lung volume, HU values, and (LAA%), demonstrating that variations in reconstruction parameters do not affect the Goddard score (GS).

**Type**	**Value**	**Goddard score (GS)**	**Both lung volume**	**HU**	**LAA%**
Slice thickness	0.6	1‐mild	1653.8	− 981	2.8
Slice thickness	0.8	0‐normal	—	− 979	—
Slice thickness	1	0‐normal	1688.7	− 981	0.6
Slice thickness	1.5	0‐normal	1696.1	− 982	0.5
Slice thickness	2	0‐normal	1706.6	− 981	0.3
Slice thickness	3	0‐normal	1719	− 979	0.2
Slice thickness	4	0‐normal	1728.5	− 973	0.1
Slice thickness	5	0‐normal	1734	− 961	0.1
Slice thickness	6	0‐normal	1735	− 954	0.1
Slice thickness	7	0‐normal	1732.9	− 926	0
Slice thickness	8	0‐normal	1730	− 908	0
Slice thickness	10	0‐normal	1717	− 834	0
FOV	250	0‐normal	—	− 980	—
FOV	260	0‐normal	—	− 981	—
FOV	270	0‐normal	1686.2	− 979	0.6
FOV	280	0‐normal	1686.7	− 980	0.6
FOV	290	0‐normal	1684.3	− 980	0.6
FOV	300	0‐normal	—	− 980	—
FOV	310	0‐normal	—	− 979	—
FOV	320	0‐normal	—	− 979	—
FOV	330	0‐normal	1687	− 979	0.6
FOV	340	0‐normal	—	− 980	—
FOV	350	0‐normal	1688.7	− 981	0.6
FOV	360	0‐normal	1689.2	− 981	0.6
FOV	370	0‐normal	1689.4	− 981	0.6
Kernel	Br 36	0‐normal	1720.9	− 1012	0.2
Kernel	Br 40	0‐normal	1715.4	− 1005	0.2
Kernel	Br 44	0‐normal	1709	− 100	0.3
Kernel	Br 48	0‐normal	1702.2	− 982	0.3
Kernel	Br 56	0‐normal	1688.7	− 982	0.6
Kernel	Br 60	0‐normal	1670.7	− 981	1.3
Kernel	Br 64	0‐normal	1658.5	− 982	2.6
Kernel	Br 68	0‐normal	1671.3	− 981	3.1
Kernel	Br 36	0‐normal	1724.8	− 1089	0.6
Kernel	Br 44	0‐normal	1715.4	− 1092	1
Kernel	Br 56	0‐normal	1685.1	− 984	1.1
Gap	0.25	0‐normal	1684.6	− 979	0.6
Gap	0.5	0‐normal	1681.9	− 979	0.6
Gap	0.75	0‐normal	1686.9	− 981	0.6
Gap	1	0‐normal	1688.7	− 981	0.6
Gap	1.25	0‐normal	1689.9	− 981	0.6
Gap	1.5	0‐normal	1689.4	− 981	0.6
Gap	1.75	0‐normal	1691.7	− 983	0.6
Gap	2	0‐normal	1690.9	− 983	0.6
Gap	2.25	0‐normal	1693.3	− 981	0.6
Gap	2.5	0‐normal	1691.8	− 981	0.6
Gap	2.75	0‐normal	1694	− 983	0.7
Gap	3	0‐normal	1694	− 981	0.7

#### 3.2.2. Case Study2

Another investigated case study presents a comprehensive quantitative assessment of emphysema using CT imaging in conjunction with the Synapse 3D Workstation (Supplementary Figure 12a–f), with a GS of 6 (mild). The axial and coronal CT scan (Supplementary Figure 12a,b) provides a segmented visualization of the lungs in a 37‐year‐old male diagnosed with emphysema, enabling a detailed evaluation of disease distribution. Color‐coded segmentation differentiates individual lobes, facilitating precise anatomical and pathological analysis. Notably, the right upper lobe (green) and right middle lobe (blue) exhibit extensive parenchymal destruction and low‐attenuation areas, hallmark features of emphysematous changes. The presence of hyperinflated regions and disruption of normal lung architecture indicates advanced disease progression, predominantly affecting the upper lobes. This heterogeneous distribution of emphysematous lesions underscores the concept of lobar‐specific susceptibility, providing insights into the pathophysiological mechanisms underlying emphysema progression.

This 3D‐rendered CT scan visualization (Supplementary Figure 12c) provides a comprehensive volumetric assessment of emphysema distribution, offering a quantitative evaluation of regional lung involvement. The segmentation of individual lobes, each assigned a distinct color, enables precise anatomical differentiation and facilitates an objective analysis of disease burden. The total lung volume is measured at approximately 6161.9 mL, with the right lung contributing 3323.7 mL and the left lung 2838.2 mL. Notably, the right upper lobe (1687.0 mL) and left upper lobe (1785.2 mL) exhibit substantial hyperinflation, a hallmark of emphysema attributed to progressive alveolar wall destruction and subsequent air trapping. In contrast, the right middle lobe demonstrates a markedly reduced volume (192.3 mL), which may be indicative of regional airway obstruction, parenchymal collapse, or compensatory volume loss due to adjacent hyperinflation. These findings highlight the heterogeneous distribution of emphysematous pathology and underscore the importance of quantitative imaging in assessing disease severity, guiding therapeutic strategies, and monitoring disease progression.

The axial CT scan (Supplementary Figure 12d) utilizes a LAA threshold of − 950 HU to quantitatively delineate emphysematous regions, categorizing them based on cluster size distribution to assess disease severity. In this case, extensive LAA clusters, particularly in the upper lobes, indicate advanced emphysema characterized by significant parenchymal destruction. The application of a color‐coded segmentation framework enables precise spatial mapping of disease distribution, facilitating a more granular evaluation of regional lung involvement. Notably, the presence of larger LAA clusters, highlighted in red and yellow, signifies extensive alveolar degradation, which may contribute to airflow obstruction and progressive pulmonary function decline. This quantitative imaging‐based approach provides a robust tool for disease staging, longitudinal assessment of therapeutic efficacy, and guiding clinical interventions in emphysema management.

This 3D‐rendered visualization (Supplementary Figure 12e) provides a detailed spatial analysis of LAA in the lungs diagnosed with emphysema. Emphysematous clusters are categorized by volume, with small clusters (blue) exceeding 2 mm^3^, moderate clusters (green) surpassing 8 mm^3^, large clusters (yellow) exceeding 65 mm^3^, and the largest clusters (red) measuring over 120 mm^3^. The widespread distribution of these clusters, particularly the extensive red‐marked regions, indicates severe alveolar destruction and significant airspace enlargement, characteristic of advanced emphysema. The concentration of large clusters across both lungs suggests substantial impairment in pulmonary function, likely contributing to compromised gas exchange and progressive airflow limitation. This quantitative 3D imaging approach provides an objective framework for assessing disease severity, monitoring progression, and informing targeted therapeutic strategies in emphysema management.

This image (Supplementary Figure 12f) presents a cumulative distribution plot of emphysematous clusters across different lung lobes, categorized by cluster volume (mm^3^). The graph employs a logarithmic scale on both axes to depict the frequency distribution of low‐attenuation clusters (LAA) relative to their size. Each lung lobe is color‐coded, revealing distinct patterns of emphysema severity and spatial distribution. The right and left upper lobes exhibit a higher prevalence of smaller clusters, whereas larger clusters are more common in the lower lobes, suggesting a differential disease progression pattern. The upper lobes demonstrate widespread microstructural damage, whereas the lower lobes exhibit more extensive alveolar destruction. The slope of each curve represents the rate of decline in cluster frequency with increasing volume, offering insights into disease heterogeneity. This quantitative analysis provides an objective framework for assessing emphysema severity, monitoring disease progression, and evaluating therapeutic efficacy.

## 4. Discussion

With the growing reliance on CT‐based quantitative metrics for COPD management, standardizing reconstruction protocols is critical to enhancing reproducibility and ensuring accurate emphysema assessment in both clinical practice and multicenter research. This study systematically evaluated the impact of CT reconstruction parameters—including kernel selection, slice thickness, FOV, and interval gaps—on EI quantification using HU‐based measurements and the GS. The findings aim to inform best practices for optimizing CT imaging protocols to improve diagnostic accuracy and facilitate cross‐institutional comparability in COPD research.

### 4.1. Slice Thickness

The results underscore the significant influence of CT slice thickness on HU values, demonstrating a consistent increase in median HU as slice thickness increases from 0.60 to 10.00 mm. This relationship, statistically confirmed through one‐way ANOVA and correlation analysis, emphasizes the need to standardize CT reconstruction parameters to ensure accurate and reproducible lung density quantification and emphysema assessment.

The observed increase in HU values with increasing slice thickness is consistent with well‐established findings in CT imaging literature. This trend is primarily attributed to the partial volume effect, a recognized artifact that arises due to signal averaging from adjacent tissues, leading to artificially elevated HU values and diminished spatial resolution in thicker slices. As a result, numerous studies have underscored the necessity of employing thinner slices to enhance the accuracy of lung density quantification, particularly in the assessment of COPD and emphysema.

For example, Bartholmai et al. [16] and Galbán et al. [17] demonstrated that thinner slices (≤ 2 mm) yield more precise HU‐based measurements, mitigating the overestimation of lung density frequently observed in thicker reconstructions. Similarly, Reeves et al. [18] reported that increasing slice thickness systematically elevates HU values, thereby influencing emphysema quantification and potentially altering disease classification. Furthermore, Boedeker et al. [7] identified significant discrepancies in emphysema scores and lung density measurements between 1 and 5 mm slices, reinforcing the imperative for standardized CT acquisition protocols to ensure consistency across clinical and research applications.

Moreover, the moderate yet statistically significant positive correlation between slice thickness and HU values (*r* = 0.40) further corroborates these findings, as illustrated in Figure 2. The statistical significance (*p* < 0.001) confirms the robustness of this association, minimizing the potential for random variation. These findings collectively emphasize the critical need for standardized CT reconstruction parameters in quantitative imaging studies, as even minor variations in HU values may have substantial implications for clinical decision‐making and disease severity classification.

While the findings of this study align with most existing literature, some research suggests that the impact of slice thickness on HU variability may be less pronounced under optimized reconstruction conditions. Uppaluri et al. [19] argued that advanced IR algorithms can mitigate partial volume effects, potentially stabilizing HU‐based measurements even in thicker slices. Similarly, Janssen et al. [20] found that advanced denoising techniques enhance measurement precision, raising the question of whether slice thickness alone should be the primary consideration in lung density quantification. However, the present study does not evaluate the influence of IR, leaving open the question of whether these advanced post‐processing methods could sufficiently counteract the HU elevation observed in thicker slices. Given the increasing reliance on CT‐based quantitative metrics for emphysema assessment, future research should investigate whether integrating IR techniques can reduce the dependence on thin‐slice acquisitions without compromising diagnostic accuracy.

From a technical perspective, these findings underscore the necessity of optimizing CT reconstruction settings to minimize artifacts and maximize spatial resolution. One key factor influencing HU variability is the partial volume effect. As slice thickness increases, the averaging of adjacent voxels leads to HU overestimation, particularly in the lung parenchyma, where low‐density emphysematous regions are interspersed with higher‐density tissues such as normal lung and blood vessels. Notably, this effect becomes most pronounced at slice thickness exceeding 5.00 mm, as demonstrated by both this study and previous research.

Moreover, a fundamental spatial resolution trade‐off must be considered. On one hand, thinner slices (≤ 2 mm) provide superior spatial resolution, minimize partial volume averaging, and preserve sharp lung structure delineation. However, this advantage comes at a cost, as thinner slices also introduce increased image noise, which can complicate HU‐based measurements. On the other hand, thicker slices (≥ 5 mm) reduce noise but simultaneously compromise spatial resolution, potentially obscuring subtle emphysematous changes that are critical for early diagnosis and disease progression monitoring. Thus, selecting an optimal slice thickness requires balancing these competing factors to ensure both image clarity and diagnostic reliability.

In addition to slice thickness, reconstruction algorithms play a crucial role in image quality and HU accuracy. Specifically, traditional filtered back projection (FBP) methods may amplify the partial volume effect, leading to greater HU overestimation in thicker slices. In contrast, newer IR techniques can enhance contrast and mitigate HU shifts, potentially improving emphysema quantification while maintaining image quality. Consequently, the choice of a reconstruction algorithm must be carefully considered when establishing standardized CT protocols for lung density assessment.

Given these complexities, further research is warranted. Future studies should explore the interaction between kernel selection, reconstruction techniques, and slice thickness to establish optimized protocols for emphysema assessment. By addressing these factors systematically, researchers and clinicians can improve the accuracy and reproducibility of HU‐based measurements, ultimately enhancing the reliability of CT‐based emphysema evaluations in both clinical and research settings.

From a practical standpoint, these findings provide critical insights for radiologists and imaging specialists involved in quantitative CT analysis of lung diseases. A key consideration is protocol standardization, particularly in slice thickness, to ensure reproducibility across institutions and improve emphysema quantification accuracy. Given the statistically significant variations in HU values across different slice thickness, maintaining a consistent slice thickness —ideally between 1.0 and 2.0 mm—is essential. Without such standardization, longitudinal patient assessments may become unreliable, affecting disease monitoring and treatment decisions.

While thinner slices enhance spatial resolution and improve lung structure delineation, they also increase image noise, necessitating careful radiation dose management. To address this, integrating low‐dose CT protocols with advanced denoising and IR techniques—such as adaptive statistical iterative reconstruction (ASIR) and model based iterative reconstruction (MBIR)—is recommended. These techniques reduce noise while preserving diagnostic quality, ensuring that high‐resolution imaging does not come at the cost of increased radiation exposure.

Additionally, AI‐driven lung density analysis tools are often optimized for slice thickness values within the 1.0–2.0 mm range, meaning deviations can introduce systematic biases in emphysema quantification. Such biases may compromise disease classification and clinical decision‐making. While some AI models incorporate correction factors, many rely on predefined reconstruction settings, reinforcing the need for consistent imaging protocols. Future research should explore whether deep‐learning‐based post‐processing techniques can enhance robustness across varying slice thickness values.

Ultimately, these findings highlight the need for collaboration among radiologists, medical physicists, and AI developers to refine CT imaging protocols. By optimizing reconstruction parameters and integrating advanced post‐processing techniques, emphysema assessment can be enhanced, leading to more reliable disease monitoring and improved clinical outcomes.

From a clinical perspective, these findings have significant implications for emphysema assessment, disease classification, and treatment planning. A key consideration is the impact on the EI, as thicker imaging slices tend to overestimate HU values, making emphysematous regions appear less hypodense. This overestimation can lead to an underestimation of disease severity, potentially affecting classification, clinical trial eligibility, and treatment decisions. Additionally, variations in slice thickness between baseline and follow‐up scans may create misleading impressions of disease progression or stability, emphasizing the need for standardized imaging protocols to ensure accurate monitoring of lung density changes over time. Furthermore, precise emphysema quantification is essential for guiding treatment decisions, including bronchodilator therapy initiation and surgical interventions such as lung volume reduction surgery. Standardizing slice thickness to ≤ 2 mm enhances diagnostic accuracy, ultimately improving treatment planning and patient outcomes.

This study reinforces the critical need to standardize CT slice thickness in quantitative lung imaging, particularly for emphysema and COPD assessment. The relationship between slice thickness and HU values highlights the effects of partial volume averaging, emphasizing the importance of protocol standardization, AI‐driven analysis, and informed clinical decision‐making. These findings underscore the need for further research in three key areas: (1) evaluating advanced reconstruction algorithms, (2) conducting longitudinal studies on emphysema progression, and (3) integrating AI for slice thickness correction. Addressing these aspects will enhance CT imaging protocols, improving reproducibility, diagnostic precision, and overall COPD management.

### 4.2. Kernels

The present study underscores the profound impact of CT reconstruction kernels on HU values, particularly in the context of quantitative lung density assessment and emphysema characterization. The observed reduction in HU values with high‐resolution (HR) kernels (Hr 36, − 1045.5 HU; Hr 44, − 1042.5 HU) compared to standard (Br) kernels (Br 44, − 998.0 HU; Br 64, − 990.0 HU, *p* < 0.05) is primarily attributed to the enhanced edge detection and noise suppression algorithms inherent in HR kernels. These findings are consistent with prior research, such as Maldonado et al. [21] and Tomica et al. [22], which demonstrated that HR kernels enhance the visualization of fine pulmonary structures but introduce greater HU variability. Furthermore, the findings align with Yoo et al. [23], who reported that HR kernels amplify noise levels, leading to increased dispersion of HU values, as reflected in the broader confidence intervals observed in Figure 4.

Despite this agreement, certain discrepancies with the existing literature merit discussion. While most studies corroborate the influence of HR kernels on HU variability, Bankier et al. [24] postulated that such variations stem not only from edge enhancement algorithms but also from differential noise‐filtering mechanisms implemented across different scanner manufacturers. This perspective suggests that the differences observed in Br 44 compared to other Br kernels in the present study may not be solely due to smoothing filter variations but could also reflect vendor‐specific algorithmic modifications. Given that such discrepancies have not been widely explored in prior research, future investigations should focus on cross‐platform standardization efforts to mitigate inter‐scanner variability.

From a technical standpoint, the findings emphasize the critical role of kernel selection in AI‐driven emphysema analysis and automated lung quantification workflows. Prior studies, including those by Chen et al. [25] and Farahat et al. [26], have highlighted that HU variability due to reconstruction settings can introduce bias into AI‐based lung segmentation models. The systematic variation in HU values observed across different kernels in this study reinforces these concerns, as it suggests that inconsistent kernel application may reduce the reliability of AI‐based emphysema classification algorithms. In line with these findings, Rajagopal et al. [27] demonstrated that variability in reconstruction parameters directly affects the performance of deep learning models for COPD phenotyping, further substantiating the need for harmonized reconstruction protocols.

However, an alternative perspective is proposed by Takahashi et al. [28], who argue that rather than enforcing strict protocol standardization, AI models should be trained on multi‐kernel datasets to enhance their robustness against HU variability. This contention raises an important question regarding the optimal approach for mitigating kernel‐induced biases in AI applications: should standardization be prioritized, or should AI‐based adaptive correction techniques be implemented? Given the growing reliance on AI for quantitative lung imaging, future research should explore whether standardization or AI‐driven correction strategies yield superior reproducibility and diagnostic accuracy in emphysema quantification and COPD assessment.

From a clinical perspective, the implications of these findings are particularly significant for HU‐based disease classification, treatment planning, and disease progression monitoring. Studies such as those by Wanger et al. [29] and Han et al. [30] have demonstrated that quantitative HU‐based emphysema scoring influences key therapeutic decisions, including bronchodilator initiation, patient eligibility for lung volume reduction surgery, and clinical trial stratification. Given that HR kernels show lower HU values, the present findings reinforce the necessity for careful kernel selection to prevent misclassification of emphysema severity. This is particularly relevant in light of Matsuoka et al. [31], who reported that HR kernels can artificially elevate HU values in fibrotic lung regions, increasing the risk of false‐positive emphysema diagnoses. Such inconsistencies further underscore the need for protocol standardization in clinical practice to ensure accurate disease classification and treatment planning.

Nevertheless, there remains debate regarding the degree of standardization required for clinical applications. While the present study supports the use of fixed kernel protocols for lung density quantification, Smith et al. [32] advocate for an adaptive approach, suggesting that reconstruction settings should be individualized based on patient characteristics rather than universally standardized. This raises a fundamental clinical question: should CT protocols be rigidly standardized to ensure reproducibility, or should they be personalized using AI‐driven correction algorithms? Answering this question will require longitudinal studies assessing the impact of HU variability on clinical decision‐making and patient outcomes.

In conclusion, this study highlights the significant impact of CT reconstruction kernel selection on HU variability, underscoring the necessity of standardized protocols for quantitative lung imaging. The observed differences in HU values between HR and Br kernels reinforce the importance of precise kernel selection to ensure reproducibility in emphysema quantification and COPD assessment. Furthermore, these findings align with previous research demonstrating that kernel‐induced HU variability can substantially influence AI‐driven lung imaging analysis, potentially compromising diagnostic accuracy. However, while standardization represents a logical solution, an alternative approach involving AI‐driven correction techniques may provide greater adaptability across different scanner settings. Therefore, future research should prioritize multi‐center validation studies to assess inter‐scanner reproducibility and evaluate whether AI‐based adjustments can effectively mitigate HU variability. Additionally, longitudinal studies are essential to determine the clinical impact of reconstruction variability on disease progression monitoring and treatment outcomes. By addressing these challenges, future investigations can contribute to optimizing quantitative CT imaging while advancing AI‐driven methodologies for precision medicine applications in COPD and emphysema management.

### 4.3. FOV

This study demonstrates that FOV variations do not significantly affect HU‐based measurements. Statistical analyses confirm this finding, showing a negligible correlation between HU and FOV (*r* = 0.00, *p* = 0.936). These findings have important technical, practical, and clinical implications, aligning with some prior studies while diverging from others in specific contexts.

From a technical standpoint, these findings confirm the reliability of quantitative CT imaging across varying FOV settings, with minimal variation in median HU values (− 989.5 to − 992.0 HU). Specifically, this consistency suggests that attenuation values remain unaffected by FOV adjustments, likely due to the intrinsic uniformity of lung parenchymal attenuation in the scanned regions.

Nevertheless, the widening of confidence intervals at larger FOVs indicates increased variability, likely due to partial volume effects and signal averaging. This observation aligns with prior studies showing that larger FOVs incorporate more peripheral soft tissue and anatomical heterogeneity, potentially compromising HU accuracy and precision. Thus, although HU values remained statistically consistent across FOV settings, the heightened variability at larger FOVs warrants careful consideration, particularly in applications requiring precise density measurements.

Furthermore, these findings support Cuneyt et al. [33], who reported that HU values in emphysema assessment remain stable within standard FOV ranges but become more variable at larger FOVs due to the inclusion of non‐parenchymal structures. However, some studies have detected minor yet statistically significant HU variations with increasing FOV, particularly when scanner calibration and reconstruction algorithms differ. As a result, these discrepancies highlight the need for standardized acquisition parameters across CT scanners.

From a practical perspective, this study provides essential insights into image acquisition standardization for clinical and research applications. Specifically, the stability of HU‐based measurements across FOV settings validates the use of different scanner protocols without compromising data integrity—an essential consideration for multi‐center studies where scanner variability is unavoidable.

Moreover, the findings confirm that radiologists and imaging specialists can adjust FOV settings based on patient size and clinical needs without compromising HU measurement accuracy. Nevertheless, the increased variability at larger FOVs reinforces the need for FOV standardization in high‐precision density assessments, such as quantitative emphysema evaluation.

Finally, while expanding the FOV enhances anatomical visualization, it should be used cautiously in quantitative CT analysis to minimize signal averaging artifacts. This caution is particularly crucial for software‐based automated emphysema quantification, where minor HU variations can influence disease classification.

Clinically, the stability of HU‐based measurements across different FOV settings reinforces the reliability of CT‐based lung density assessments for emphysema diagnosis and progression monitoring. Since small HU variations can significantly impact disease staging and treatment decisions, these findings provide confidence that FOV adjustments do not introduce systematic bias into lung density measurements.

Furthermore, the negligible correlation between HU and FOV is particularly relevant for longitudinal studies, where patients undergo repeated scans with varying FOV settings. These findings confirm that differences in FOV between baseline and follow‐up imaging do not compromise HU‐based disease monitoring, thereby improving the consistency and reliability of patient evaluations over time.

Moreover, the study’s findings support the integration of quantitative CT biomarkers into routine clinical practice by demonstrating the robustness of HU values across different imaging protocols. This stability is essential for developing AI‐driven diagnostic models, which require consistent input parameters to ensure accurate disease classification and risk stratification.

The findings of this study align with prior research demonstrating that HU values remain stable across varying FOV settings. Studies by Huaiyu et al. [34] similarly found no statistically significant variation in HU‐based measurements across different FOVs, reinforcing the reliability of quantitative CT imaging for lung density assessments. Moreover, these studies emphasize that attenuation values in homogeneous lung regions are largely unaffected by FOV changes, further supporting the conclusion that adjustments in scan parameters do not compromise measurement accuracy. Additionally, the observed increase in variability at larger FOVs, attributed to partial volume effects, aligns with previous findings highlighting the impact of peripheral anatomical structures on signal averaging. Finally, the stability of HU values across different FOV settings underscores the robustness of CT‐based biomarkers in multi‐center studies, ensuring consistency in longitudinal disease monitoring and AI‐driven diagnostic applications.

While this study’s findings align with previous research, some studies have reported subtle yet statistically significant variations in HU values with increasing FOV. For example, Anne et al. [35] suggested that minor HU shifts may result from differences in scanner calibration, reconstruction algorithms, or patient positioning. Although the present study did not detect systematic HU changes, these discrepancies underscore the potential impact of scanner‐specific parameters on quantitative CT analysis. Moreover, variations in reconstruction kernels and dose modulation techniques among different scanner manufacturers have been reported to introduce inconsistencies in attenuation measurements. These findings suggest that while FOV adjustments may not significantly affect HU values in controlled settings, variability across scanners and imaging protocols remains a concern. Thus, further research is necessary to determine whether standardizing scanner‐specific parameters can fully mitigate minor HU fluctuations associated with FOV changes.

Overall, this study provides strong evidence that FOV adjustments do not significantly affect HU‐based measurements, reinforcing the reliability of CT‐based lung density quantification. However, the increased variability observed at larger FOVs highlights the need for careful protocol optimization in quantitative imaging applications. These findings further support standardization efforts in multi‐center studies and clinical practice, ensuring consistent and reproducible HU‐based measurements for emphysema evaluation and disease monitoring.

### 4.4. Gap

The findings of this study indicate that HU‐based measurements remain stable across different reconstruction gap sizes, with no statistically significant trend observed. Although the median HU values increased slightly from − 997.25 HU (0.25 mm) to − 994.25 HU (3 mm), this variation was not statistically significant (*p* = 0.966). Additionally, the IQR remained stable, reinforcing the robustness of HU‐based measurements across different gap sizes. These results suggest that gap size variations within the examined range do not systematically affect HU values, supporting the reliability and reproducibility of CT imaging. However, the wide confidence intervals observed in Figure 6a highlight inherent variability, which may stem from image noise, scanner calibration differences, or reconstruction algorithms. Future research should investigate these potential sources of variation and assess whether specific conditions, such as tissue composition or contrast agent use, influence HU stability.

The correlation analysis (Table 7) revealed a weak positive association (*r* = 0.111) between gap size and HU values, but this relationship was not statistically significant (*p* = 0.125). Similarly, the scatter plot (Figure 6b) demonstrated substantial dispersion of data points around the regression line, reinforcing the lack of a meaningful correlation. These findings suggest that gap size alone does not substantially impact HU values. However, the observed data variability underscores the importance of controlling confounding factors, such as scanner settings, radiation dose, and patient‐specific anatomical variations, in future studies. While our results support the reliability of HU‐based imaging, they also emphasize the need for standardized imaging protocols to minimize inconsistencies, particularly in multi‐center studies and AI‐driven radiomics applications.

These results align with previous studies indicating that minor adjustments in reconstruction parameters, such as gap size, do not significantly alter HU values [36]. However, some researchers have reported statistically significant HU changes with variations in slice thickness and reconstruction kernels [37, 38]. The discrepancy between our findings and these studies may stem from differences in scanner models, post‐processing algorithms, or anatomical regions analyzed. For instance, Zhou et al. [37] utilized a different CT scanner model and kernel settings, which may have introduced systematic HU variations. Furthermore, lung imaging is particularly sensitive to reconstruction settings, as noted by Kim et al. [39], who found that even small modifications in post‐processing parameters influenced HU values in lung tissue characterization. Our results, however, suggest that gap size alone does not significantly alter HU values, reinforcing the robustness of CT‐based density assessments. Future studies should explore whether specific anatomical regions or pathological conditions exhibit greater HU sensitivity to reconstruction settings, particularly in lung and bone imaging, where subtle HU variations may affect diagnostic accuracy.

While our findings indicate that HU values remain stable across different gap sizes, practical trade‐offs in clinical imaging must be considered. Narrower gap sizes (e.g., 0.25 mm) enhance spatial resolution but may increase image noise, whereas wider gaps (e.g., 3 mm) reduce computational demands and improve image smoothness but may obscure fine anatomical details. These trade‐offs are particularly relevant in applications such as lung nodule assessment, bone mineral density evaluation, and tumor characterization, where subtle HU differences can impact diagnosis. Future research should determine the optimal balance between spatial resolution, noise reduction, and computational efficiency for different clinical indications.

From a clinical and radiological perspective, these findings have significant implications for optimizing CT protocols. Given that HU values remain stable within the tested gap size range, radiologists and imaging specialists can adjust reconstruction settings to prioritize either image quality or computational efficiency without compromising diagnostic accuracy. Additionally, the robustness of HU‐based measurements supports standardization efforts, particularly in longitudinal studies and multi‐center clinical trials, where consistency is crucial. Some researchers have emphasized the need for strictly standardized reconstruction parameters to minimize variability [40]. While standardization remains essential, our findings suggest that moderate flexibility in gap size selection does not introduce significant biases, allowing for protocol adjustments based on clinical requirements.

Moreover, these findings induce direct implications for AI‐driven imaging analyses. The increasing use of machine learning and deep learning models in CT diagnostics necessitates consistent and reproducible HU values to ensure accurate predictions. The demonstrated stability of HU values suggests that minor variations in reconstruction gap size are unlikely to introduce significant biases in AI‐based disease classification. However, some studies have raised concerns that even subtle HU fluctuations could impact AI model performance, particularly in radiomics‐based feature extraction [41]. Future research should explore whether AI algorithms require adaptation to accommodate minor HU variations across different CT protocols and investigate whether deep learning‐based noise reduction techniques could further enhance HU stability.

This study provides strong evidence that HU values remain stable across different reconstruction gap sizes, with no statistically significant effect observed. The findings support the reliability and reproducibility of quantitative CT imaging. However, while gap size alone does not appear to introduce significant HU variability, the wide confidence intervals highlight potential influences from image noise, scanner calibration, and reconstruction algorithms. Additionally, clinical trade‐offs in gap size selection must be considered, particularly in applications requiring high spatial resolution. Future research should explore the interaction between reconstruction parameters, scanner variability, and specific disease conditions to further refine CT standardization protocols and optimize quantitative imaging accuracy.

Despite the robustness of these findings, certain limitations must be acknowledged. First, the sample size may limit the generalizability of the results, particularly across diverse patient populations with varying anatomical structures and pathological conditions. Second, this study was conducted using a single CT scanner model, which may not fully account for inter‐scanner variability in HU‐based measurements. Prior research has indicated that different scanner manufacturers may introduce slight HU variations due to differences in detector calibration and reconstruction software [42]. Therefore, future multi‐center studies using different scanner models and larger patient cohorts are needed to confirm these findings in broader clinical settings. Lastly, while this study focused on HU stability in a controlled experimental setting, future research should assess how reconstruction settings interact with disease‐specific imaging, such as pulmonary fibrosis or osteoporosis assessment, where HU values play a critical diagnostic role.

### 4.5. Goddard Score (GS)

The findings indicate that variations in FOV, kernel settings, and gap size do not significantly affect the GS, suggesting that these reconstruction parameters do not introduce structural distortions in pulmonary imaging. Statistical analysis confirmed that differences in these parameters resulted in minimal variation in lung attenuation values and qualitative assessment (*p* > 0.05). This aligns with previous literature showing that FOV primarily affects image resolution rather than lung attenuation values, while kernel selection influences image sharpness without fundamentally altering lung assessment. Similarly, studies suggest that variations in gap size have negligible effects on lung structure evaluation, reinforcing the robustness of CT imaging protocols under standard clinical conditions.

However, some studies indicate that kernel selection may impact emphysema quantification due to noise and edge enhancement, while larger gap sizes could slightly underestimate emphysema severity. Additionally, the results highlight that ultra‐thin slices (0.6 mm) improve the detection of subtle lung pathology, leading to a mild increase in GS. This supports prior findings that high‐resolution CT enhances sensitivity to early‐stage lung abnormalities by reducing partial volume effects. However, increased noise at thinner slice thickness may complicate image interpretation, a limitation not quantitatively assessed in this study. Moreover, some evidence suggests ultra‐thin slices may not significantly alter GS unless focal emphysematous changes are present, indicating that their impact may depend on disease distribution and severity.

Overall, these findings support the reliability of CT imaging across a range of reconstruction parameters while emphasizing the need to optimize slice thickness to balance diagnostic sensitivity and image clarity. Future research should further explore the effects of these parameters on quantitative lung assessments, incorporating AI‐based texture analysis to enhance early disease detection beyond qualitative scoring methods.

This study highlights the impact of CT reconstruction parameters on EI quantification, emphasizing the need for standardized imaging protocols in COPD assessment. However, several limitations should be considered. The relatively small sample size (31 subjects) may limit the generalizability of these findings, particularly across populations with varying disease severities and anatomical differences. Additionally, using a single CT scanner model may not fully account for inter‐scanner variability, which could affect reproducibility in multi‐center studies. While this study systematically evaluated slice thickness, kernel selection, FOV, and reconstruction gaps, it did not assess their interactions with advanced iterative reconstruction techniques. Furthermore, the effect of radiation dose variations on emphysema quantification—an important factor in low‐dose CT protocols—remains unexamined.

Despite these limitations, the findings confirm the reliability of the GS and HU‐based emphysema quantification, with minimal structural distortions observed in pulmonary imaging. The results further suggest that ultra‐thin slices improve the detection of subtle lung pathology, although increased noise may complicate interpretation. Optimizing slice thickness is crucial for balancing diagnostic sensitivity and image clarity, while AI‐driven texture analysis is a promising tool for improving quantitative emphysema assessment. Future multi‐center studies with larger cohorts should validate the reproducibility of these findings across different scanners and imaging protocols while exploring AI‐based methods to enhance standardization and reduce variability in quantitative imaging.

## 5. Conclusion

This study underscores the critical influence of CT reconstruction parameters on EI quantification in COPD assessment, emphasizing the need for standardized imaging protocols to ensure reliable quantification. The findings demonstrate that variations in FOV, kernel selection, and reconstruction gaps have minimal impact on the GS, reinforcing the robustness of CT‐based emphysema evaluation. However, ultra‐thin slices (0.6 mm) enhance the detection of subtle lung pathology, though increased noise may complicate interpretation. While HU‐based emphysema quantification remains valuable, optimizing reconstruction settings—particularly slice thickness—is crucial for balancing diagnostic sensitivity and image clarity.

Further research should refine AI‐driven analysis techniques to improve emphysema detection and cross‐institutional comparability in COPD management. Multi‐center studies with larger cohorts are needed to validate these findings and optimize reconstruction algorithms to reduce noise and enhance diagnostic precision in quantitative lung imaging.

## Ethics Statement

The authors followed the ethics of research, approved, and consented to participate in this study.

## Consent

The authors approved this version of the manuscript for publication.

## Conflicts of Interest

The authors declare no conflicts of interest.

## Author Contributions

Dr. Ahmed AG El‐Shahawy conceived the study and designed the research framework. Dr. El‐Shahawy and Rahma Saad Mohamed Mohamed developed the methodology. Rahma Saad Mohamed conducted the experimental work. Data analysis and interpretation were performed by Dr. El‐Shahawy and Rahma Saad Mohamed. Both authors contributed to manuscript drafting. Dr. El‐Shahawy, Rahma Saad Mohamed, and Ahmed Sayed Abd Bassset El Basset critically reviewed and revised the manuscript for intellectual content. All authors approved the final version for publication and assume full responsibility for the accuracy, integrity, and scientific validity of the work, ensuring that any concerns are properly addressed.

## Funding

No funding was received for this manuscript.

## Supporting information


**Supporting Information** Additional supporting information can be found online in the Supporting Information section. Supplementary Figure 1. ROI placement over emphysematous regions in lung window used for attenuation measurements. Symmetric placement was used to standardize HU quantification across subjects. Supplementary Figure 2. Lung segmentation in axial, sagittal, and coronal planes using Synapse 3D workstation. This automated segmentation minimizes observer variability in emphysema evaluation. Supplementary Figure 3. Labeling of low attenuation areas (LAA) below − 950 HU in axial, sagittal, and coronal views. These regions correspond to emphysematous tissue loss. Supplementary Figure 4. 3D volume rendering of segmented lungs for quantitative emphysema assessment. This enables volumetric comparison of affected vs. preserved lung tissue. Supplementary Figure 5. Cluster‐based 3D volume rendering of LAA regions. LAA clusters are volumetrically grouped for improved spatial analysis of emphysema severity. Supplementary Figure 6. Histogram‐based attenuation analysis of lung parenchyma. The distribution of voxel HU values supports quantitative density assessment. Supplementary Figure 7. Anatomical division of lung into upper (aortic arch), middle (carina), and lower (diaphragm) sections for regional GS assignment. Supplementary Figure 8. Representative CT axial images reconstructed at different slice thickness: (a) 0.6 mm, (b) 5 mm, (c) 10 mm. thinner slices provide higher spatial resolution; thicker slices demonstrate blurring and partial volume effects. Supplementary Figure 9. Representative axial images with different reconstruction kernels: (a) Br 48, (b) Hr 44, (c) Hr 64. High‐resolution kernels improve edge definition but may introduce noise. Supplementary Figure 10. CT axial images reconstructed using varying FOV settings: (a) 250 mm, (b) 290 mm, (c) 360 mm. HU values remain stable across different FOVs, supporting robustness of density quantification. Supplementary Figure 11. CT axial images reconstructed with different reconstruction gaps: (a) 0.25 mm, (b) 2 mm, (c) 3 mm. Minimal variation in HU observed, indicating measurement stability. Supplementary Figure 12. Multiplanar and volumetric CT analysis of emphysema in a representative patient: (a) Segmented axial CT image showing LAA regions. (b) Color‐coded lobe segmentation. (c) Volumetric emphysema quantification. (d) Cluster volume‐based LAA categorization. (e) 3D spatial distribution of LAA clusters. (f) Cumulative cluster distribution by lung lobe. These views facilitate granular analysis of emphysema burden and heterogeneity.

## Data Availability

The authors emphasize the availability of data and materials.
