# Supplementary material for: The Impact of CT Reconstruction Parameters on Emphysema Index Quantification, HU‐Based Measurements, and Goddard Score in COPD Assessment: A Prospective Study
Source: Int J Biomed Imaging. 2026 Jan 9;2026:7436511. doi: 10.1155/ijbi/7436511 (PMC12789179; doi:10.1155/ijbi/7436511)
Supplement: Supplementary file 1 — Supporting Information Additional supporting information can be found online in the Supporting Information section. Supplementary Figure 1. ROI placement over emphysematous regions in lung window used for attenuation measurements. Symmetric placement was used to standardize HU quantification across subjects. Supplementary Figure 2. Lung segmentation in axial, sagittal, and coronal planes using Synapse 3D workstation. This automated segmentation minimizes observer variability in emphysema evaluation. Supplementary Figure 3. Labeling of low attenuation areas (LAA) below − 950 HU in axial, sagittal, and coronal views. These regions correspond to emphysematous tissue loss. Supplementary Figure 4. 3D volume rendering of segmented lungs for quantitative emphysema assessment. This enables volumetric comparison of affected vs. preserved lung tissue. Supplementary Figure 5. Cluster‐based 3D volume rendering of LAA regions. LAA clusters are volumetrically grouped for improved spatial analysis of emphysema severity. Supplementary Figure 6. Histogram‐based attenuation analysis of lung parenchyma. The distribution of voxel HU values supports quantitative density assessment. Supplementary Figure 7. Anatomical division of lung into upper (aortic arch), middle (carina), and lower (diaphragm) sections for regional GS assignment. Supplementary Figure 8. Representative CT axial images reconstructed at different slice thickness: (a) 0.6 mm, (b) 5 mm, (c) 10 mm. thinner slices provide higher spatial resolution; thicker slices demonstrate blurring and partial volume effects. Supplementary Figure 9. Representative axial images with different reconstruction kernels: (a) Br 48, (b) Hr 44, (c) Hr 64. High‐resolution kernels improve edge definition but may introduce noise. Supplementary Figure 10. CT axial images reconstructed using varying FOV settings: (a) 250 mm, (b) 290 mm, (c) 360 mm. HU values remain stable across different FOVs, supporting robustness of density quantification. Suppleme [file IJBI-2026-7436511-s001.docx]

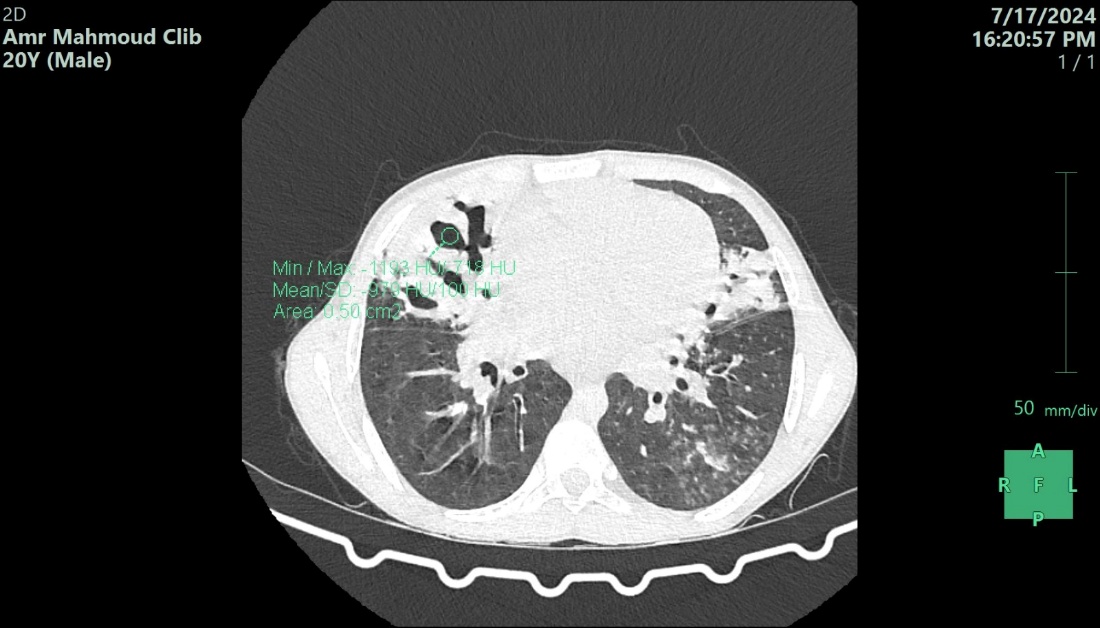


a


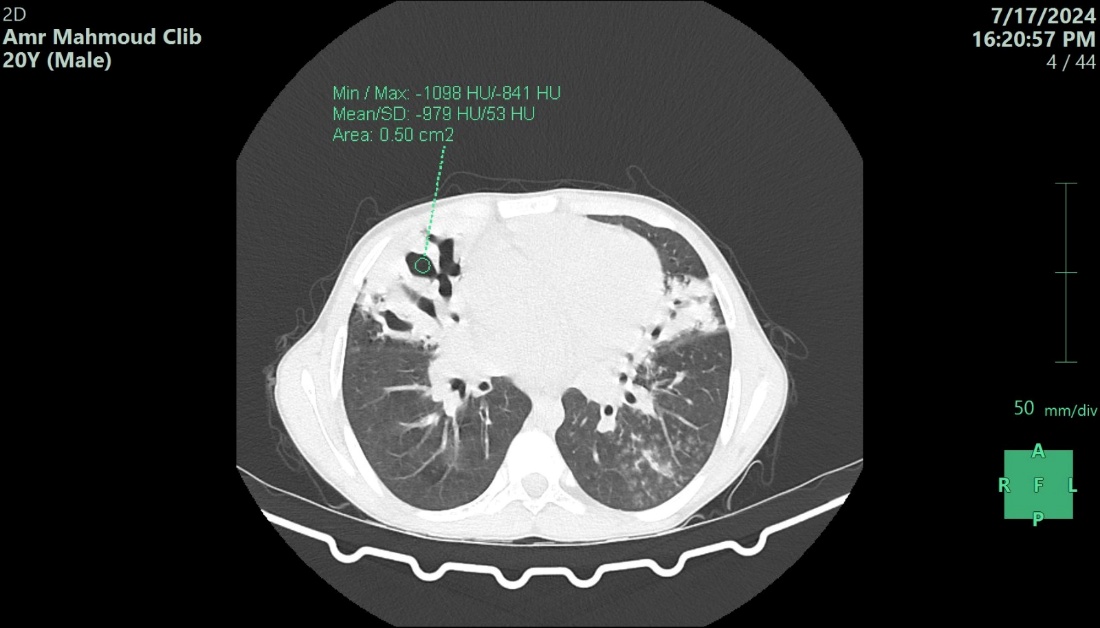


b


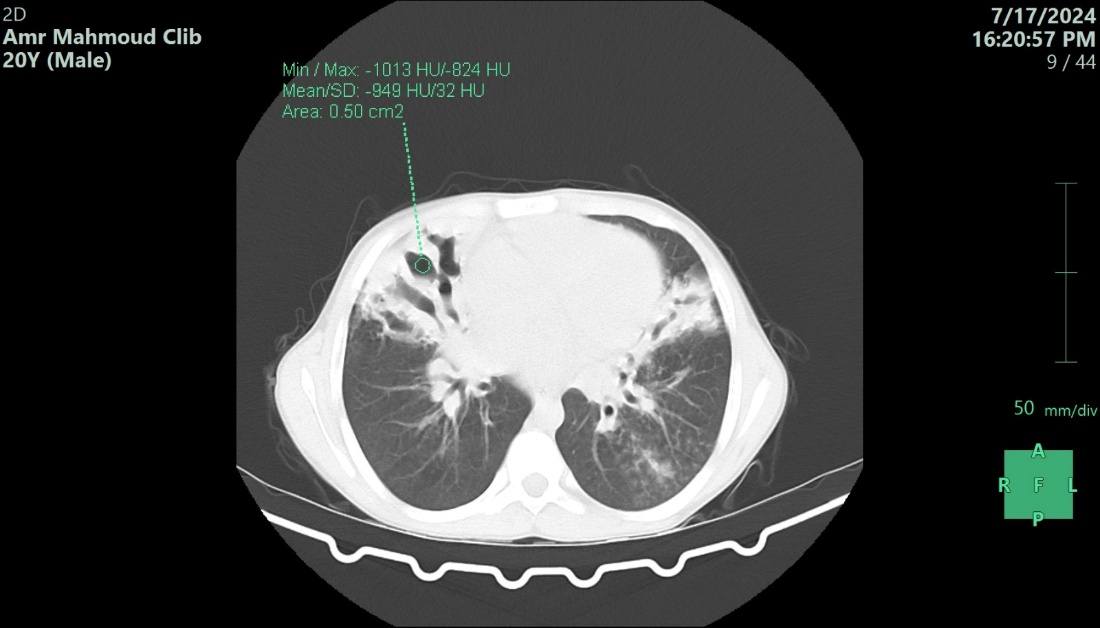


c

Supplementary Fig.1


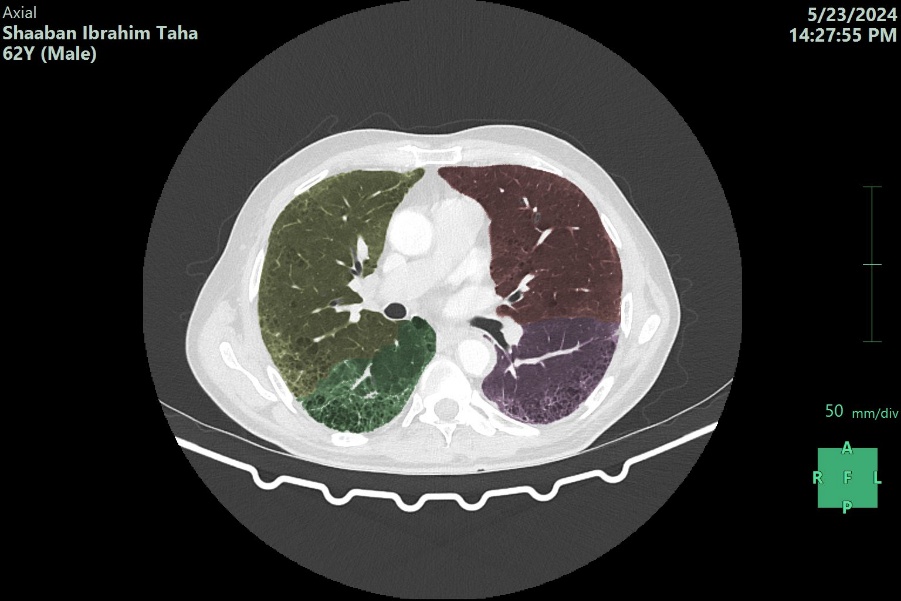


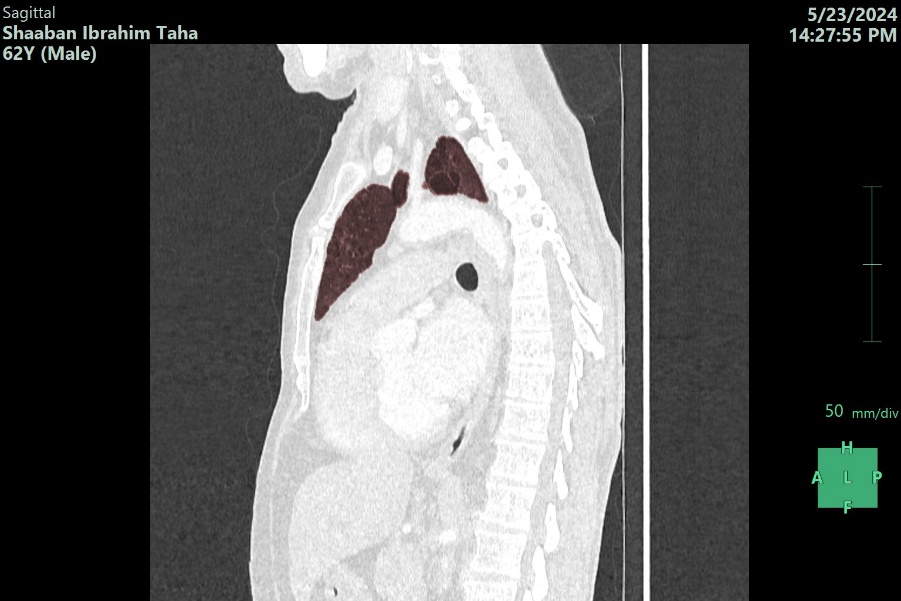


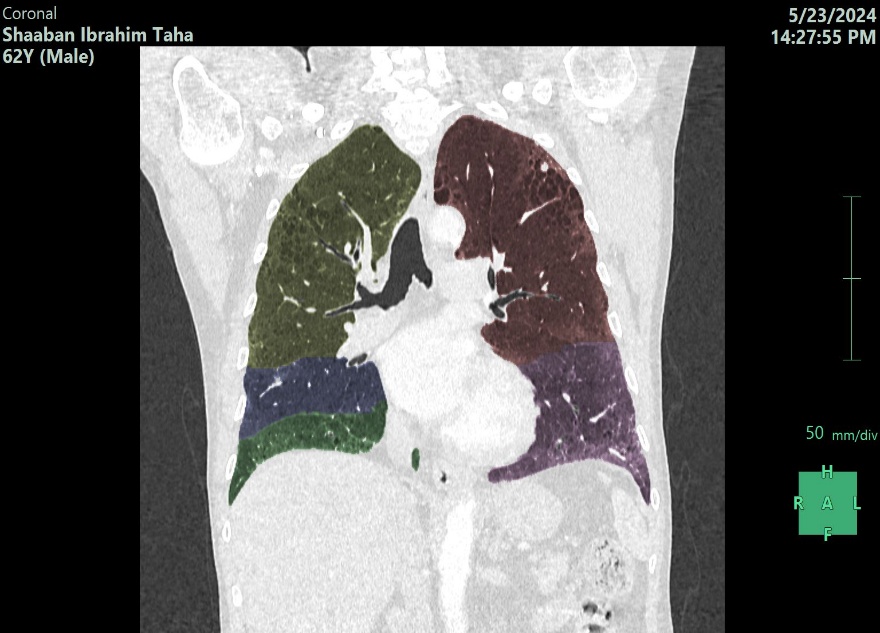


Supplementary Fig.2


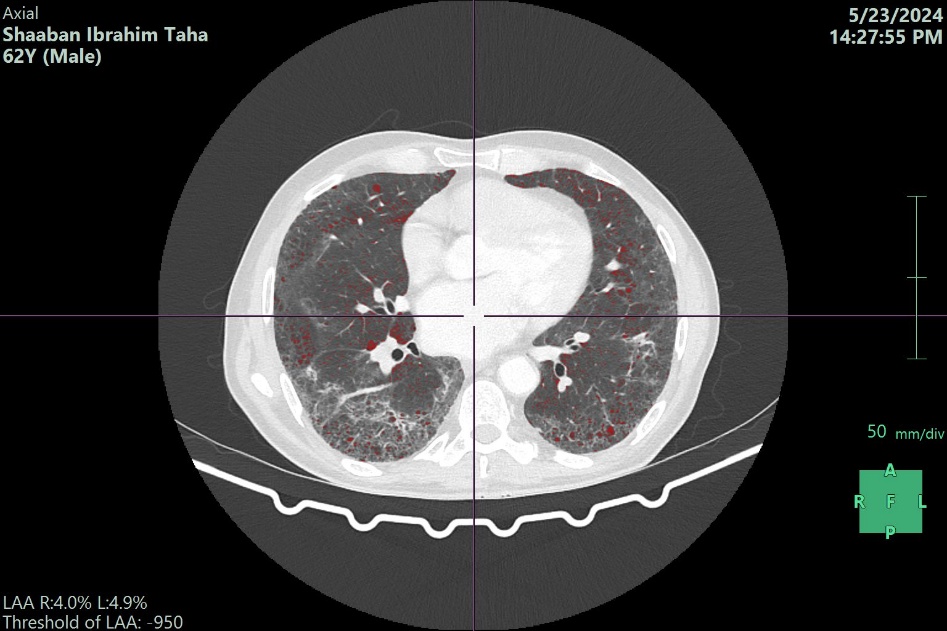


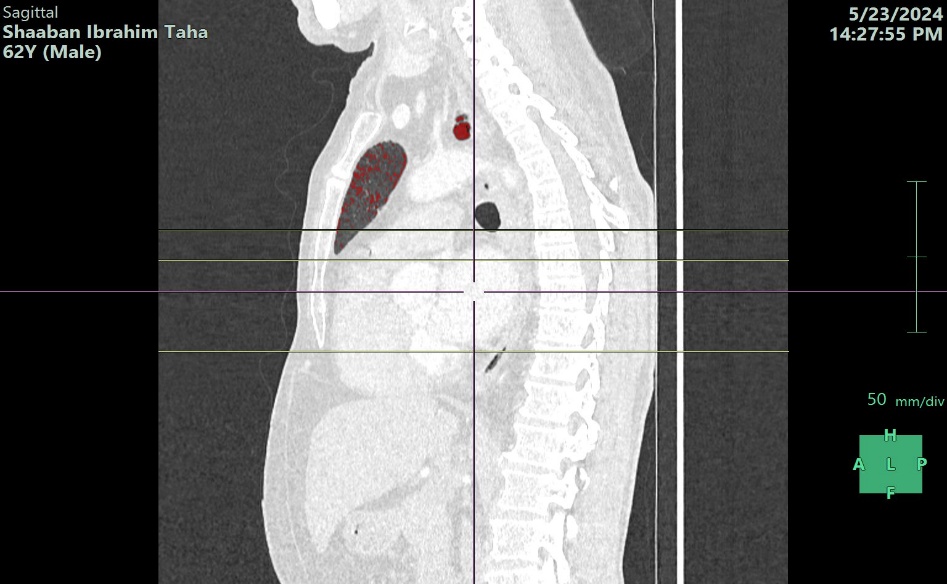


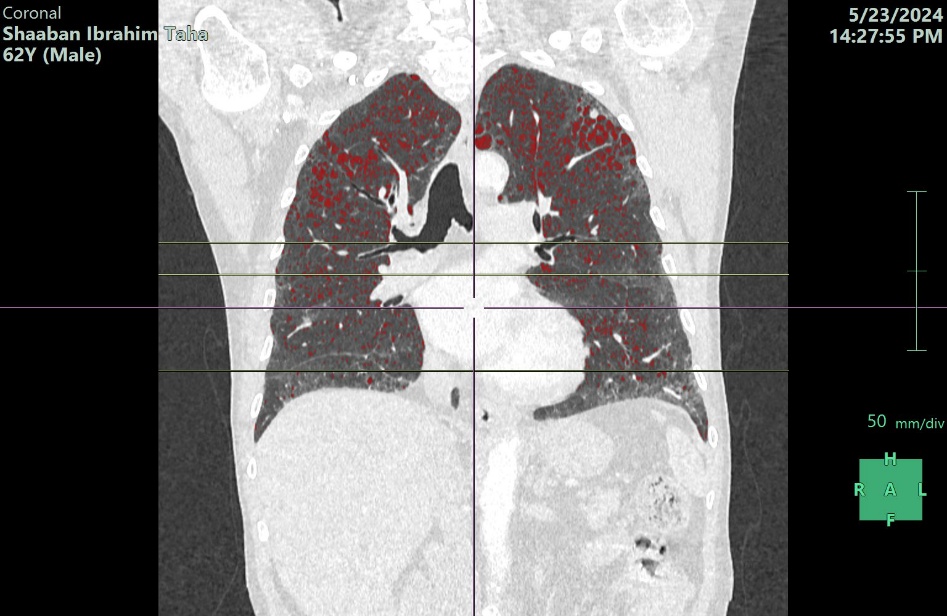


Supplementary Fig.3


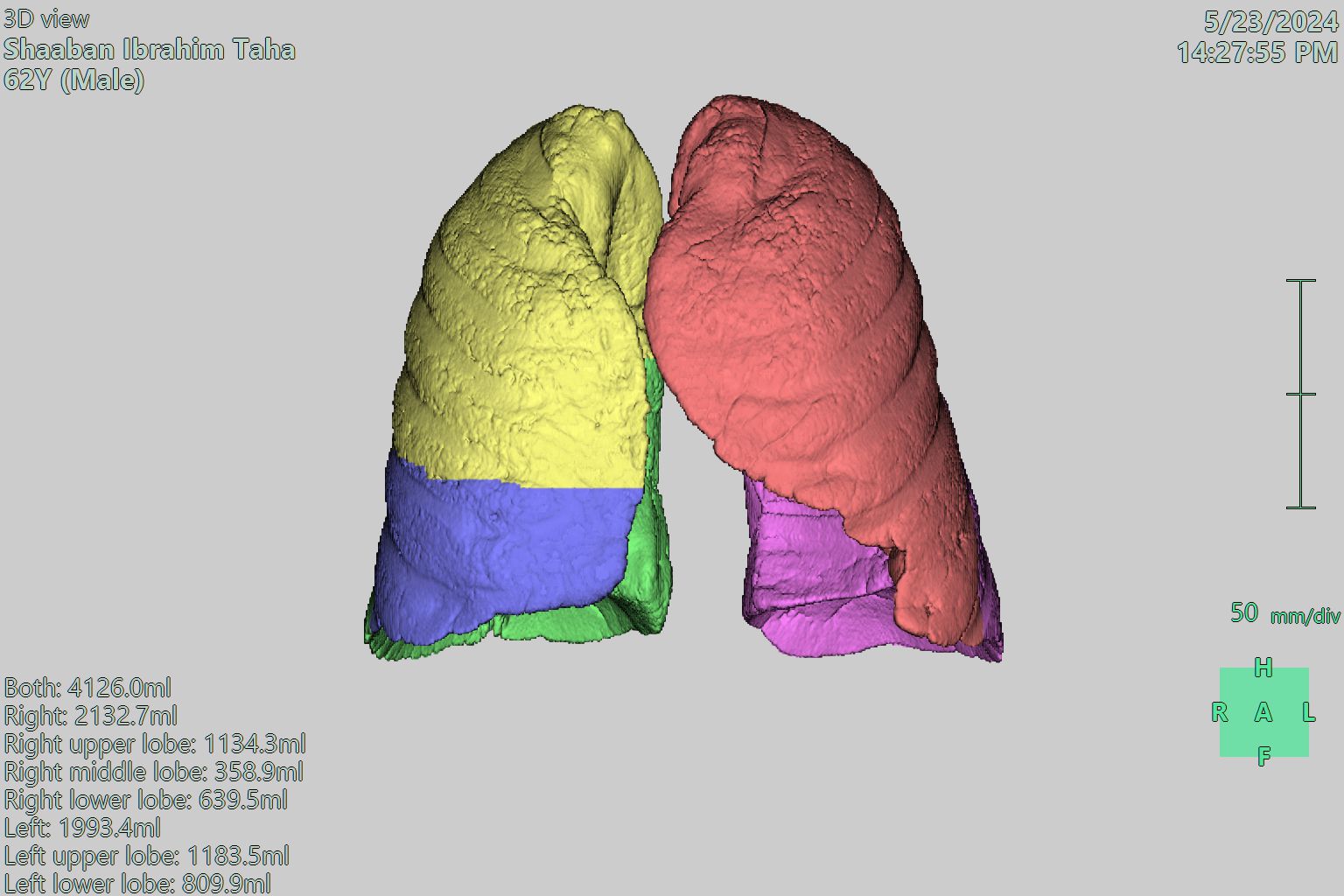

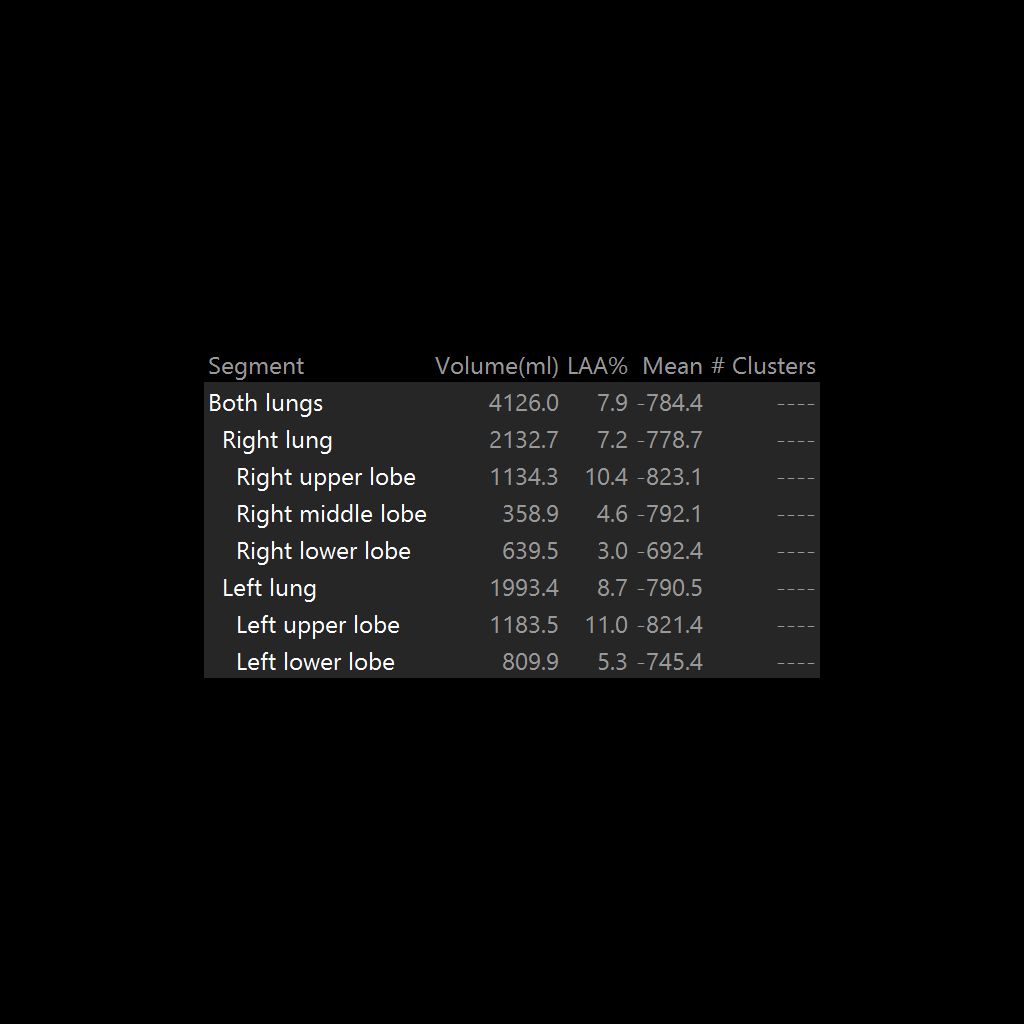


Supplementary Fig.4


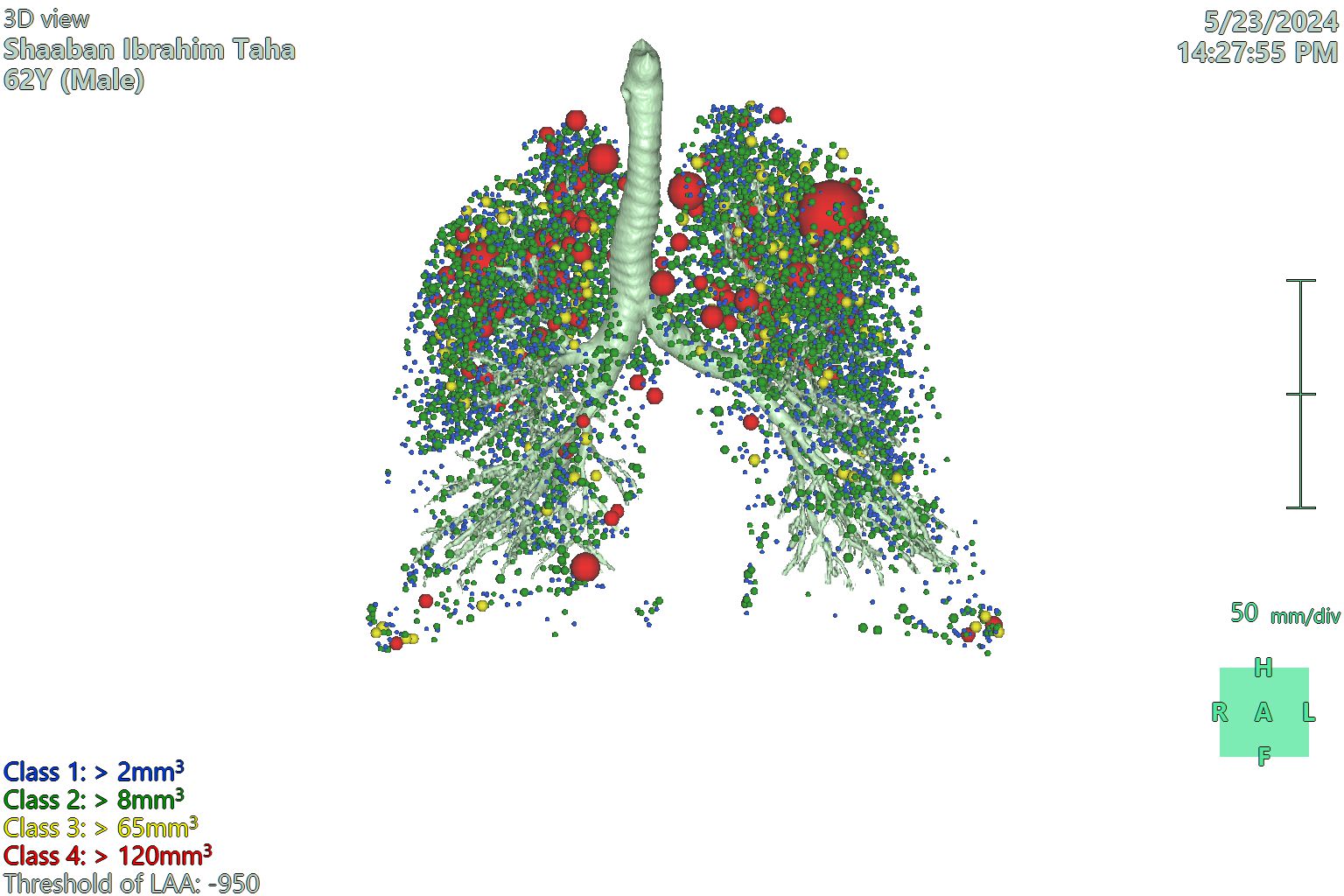


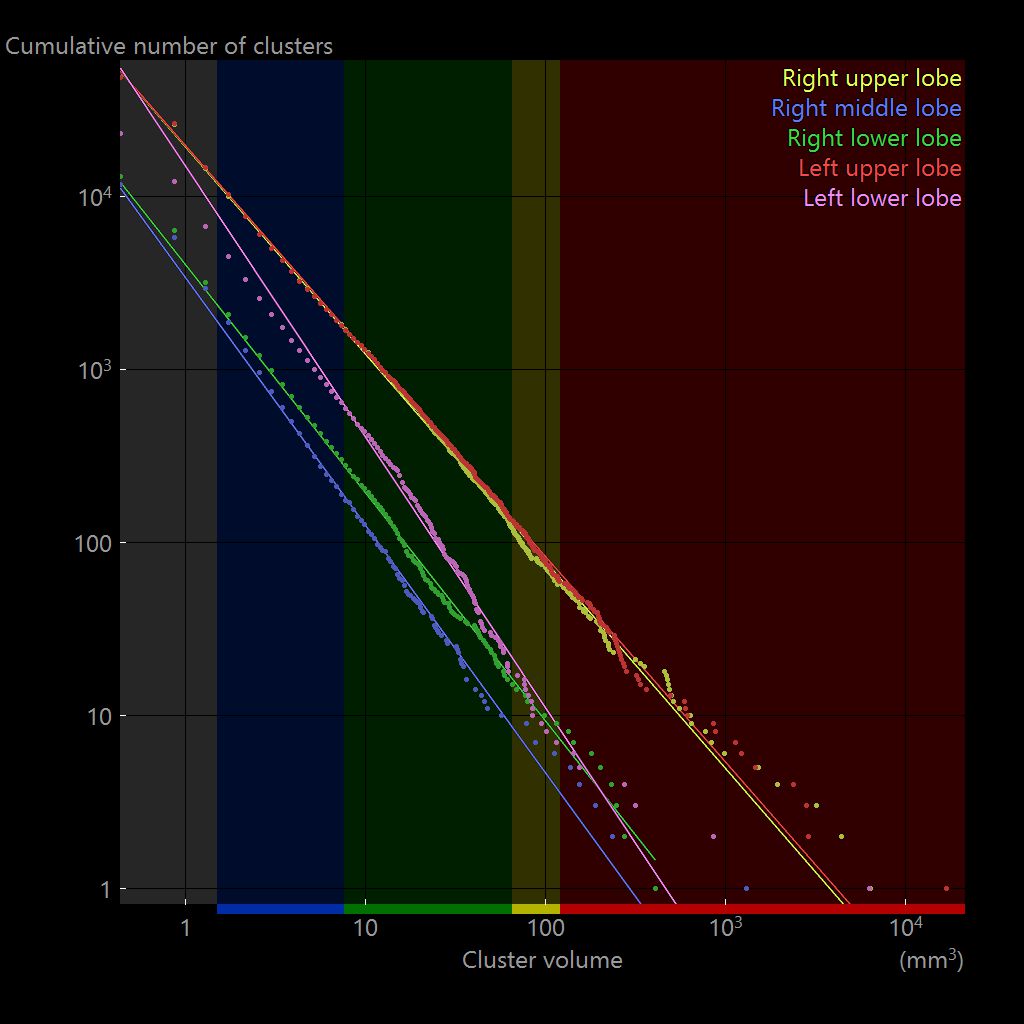


Supplementary Fig.5


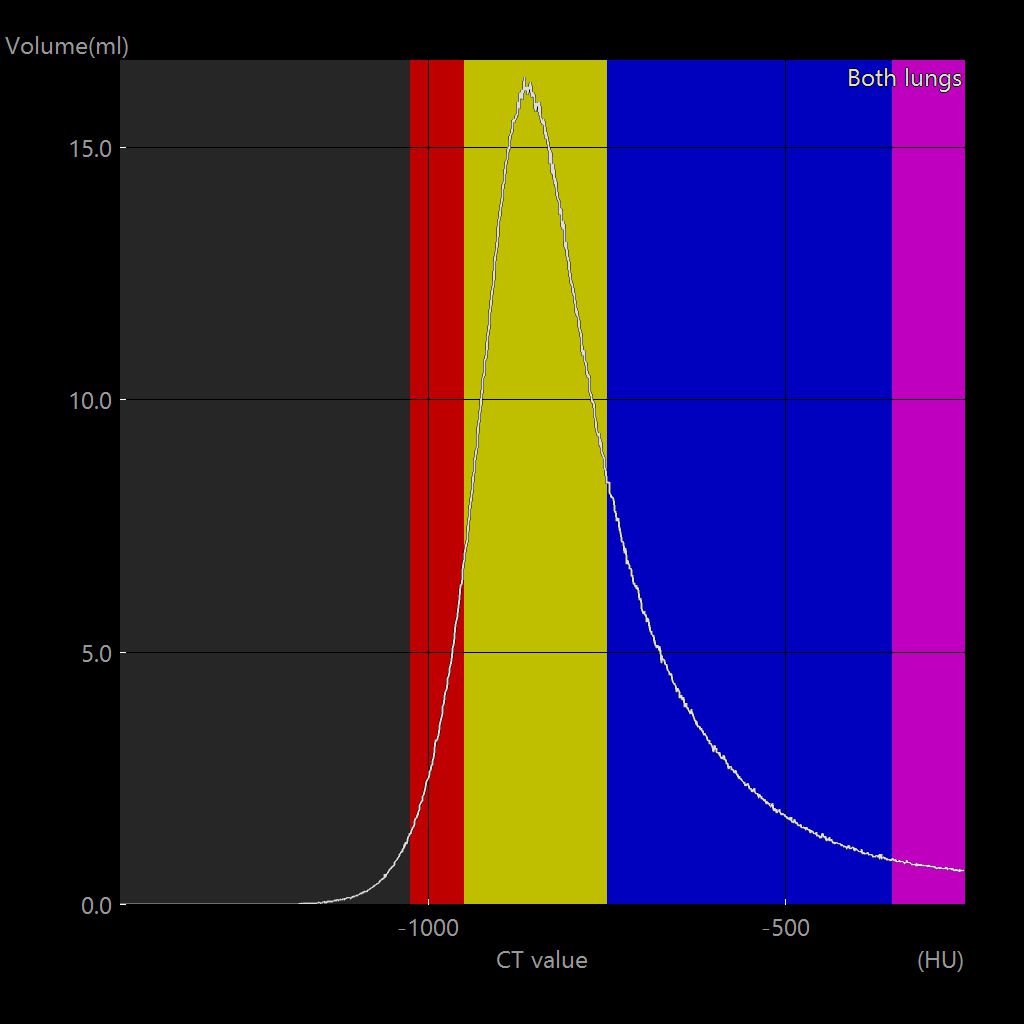


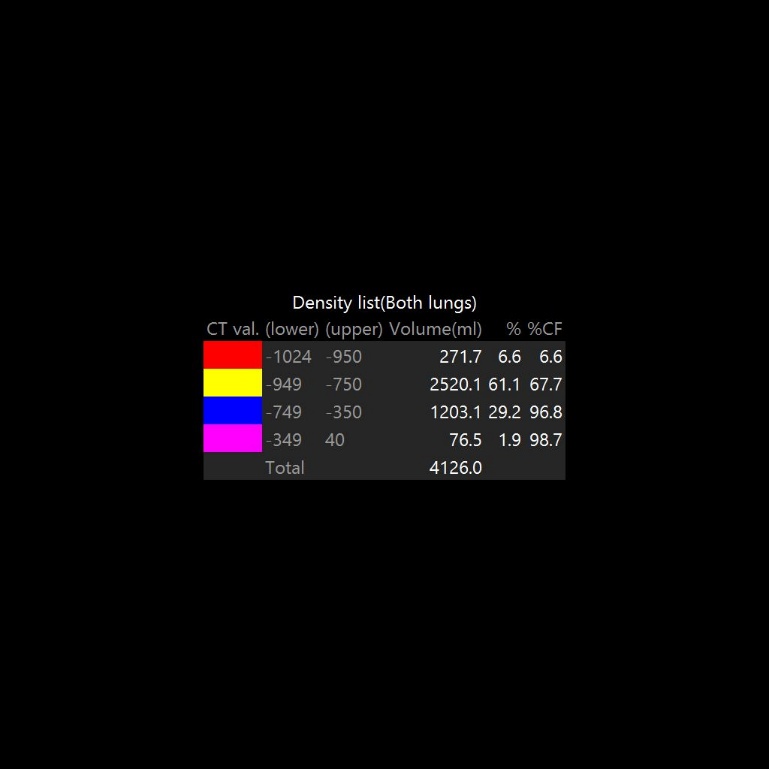


Supplementary Fig.6


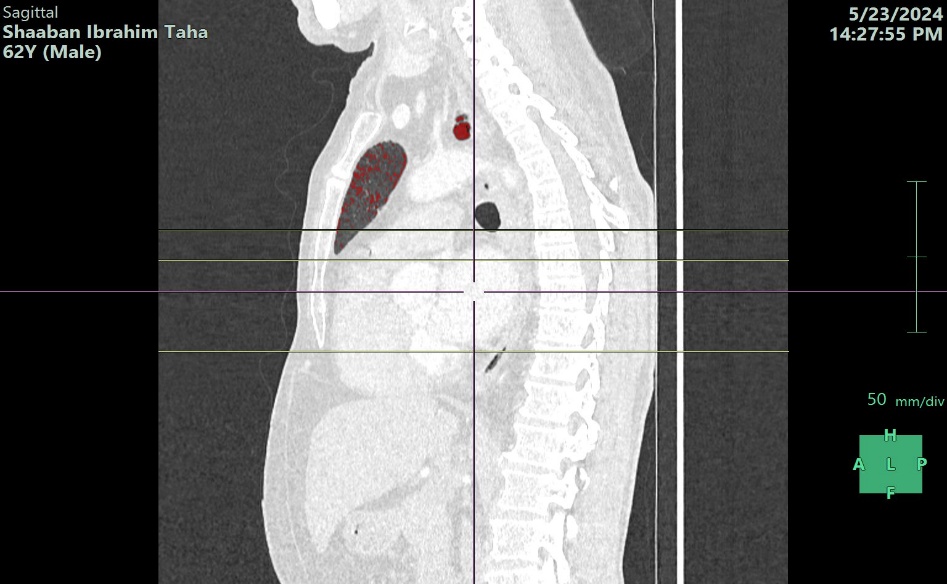


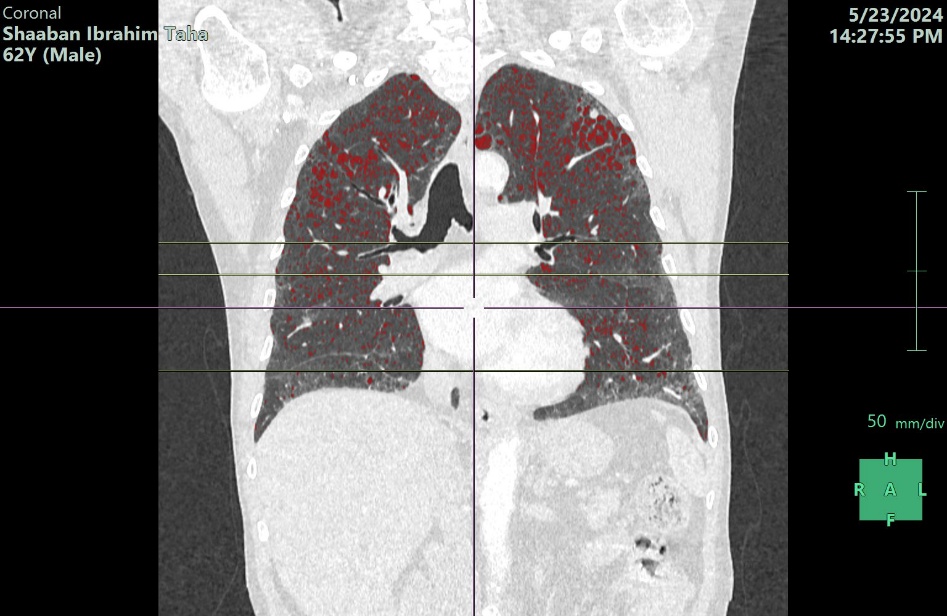


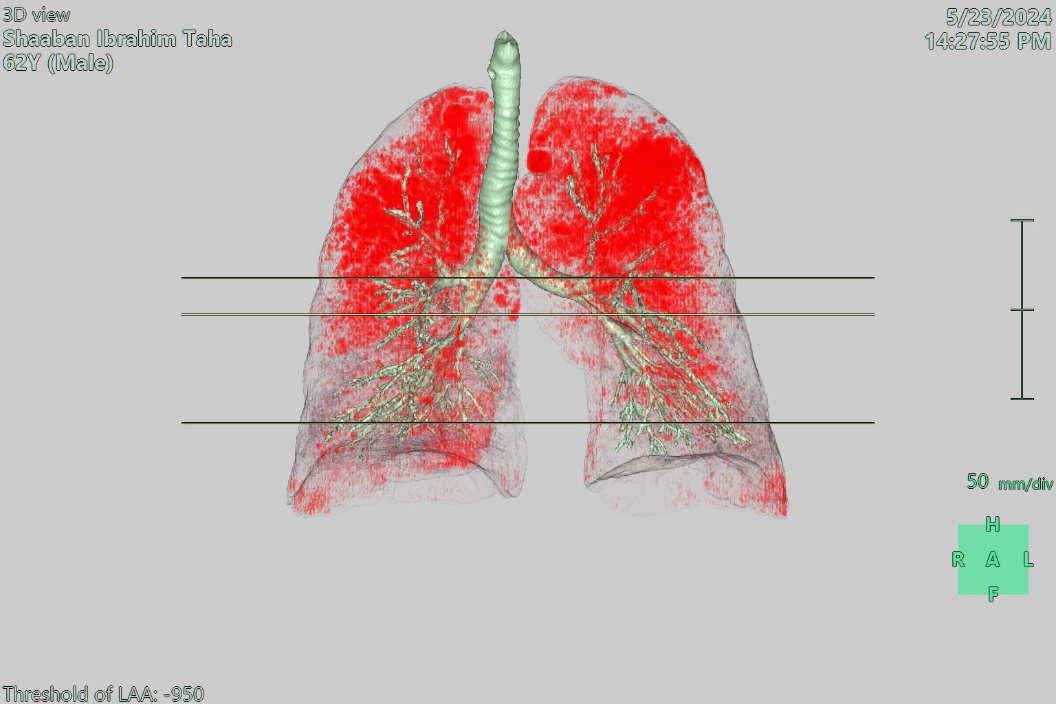


Supplementary Fig.7


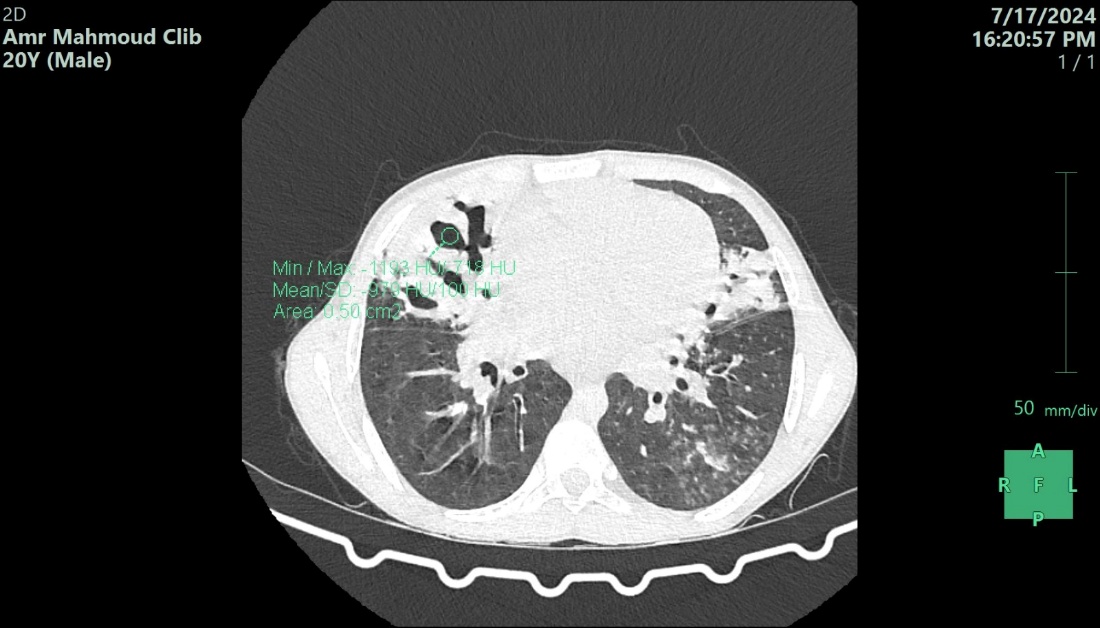


0.6mm


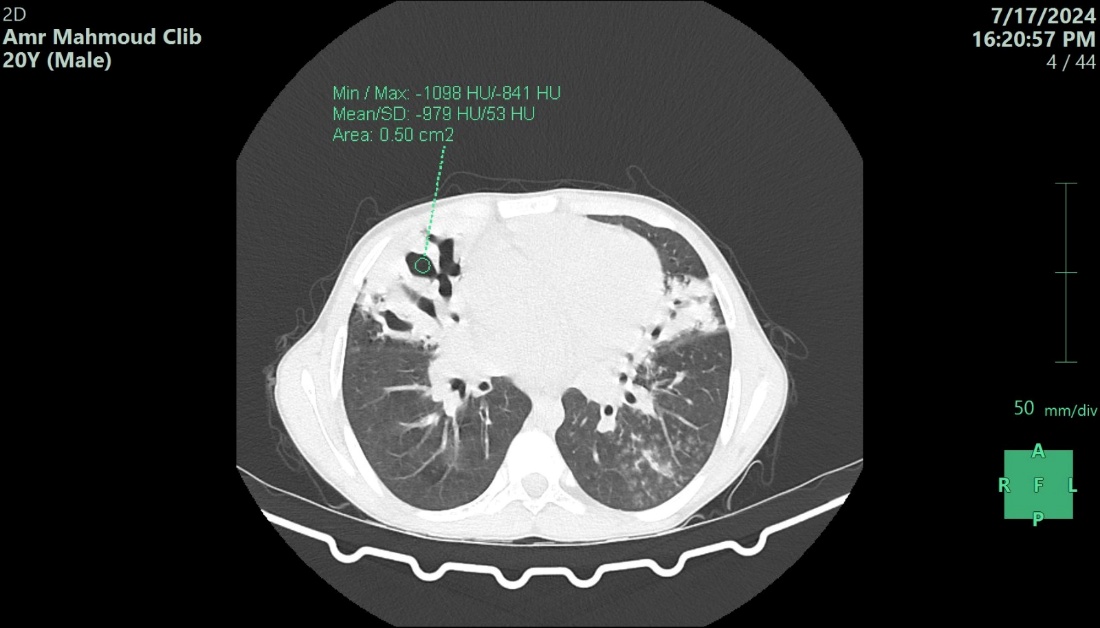


5mm


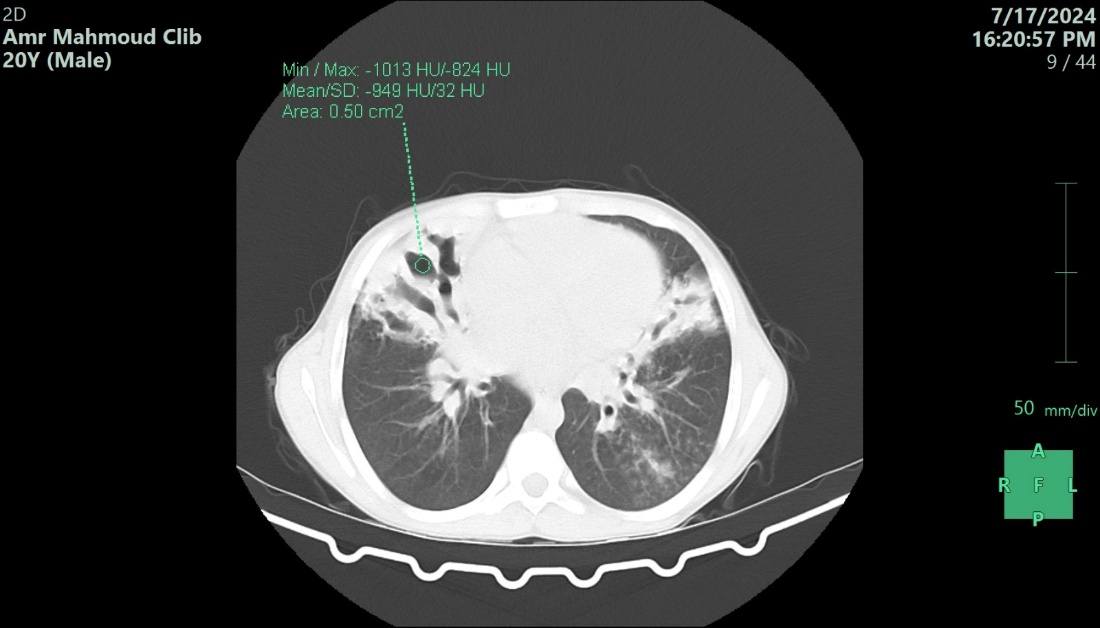


10mm

Supplementary Fig.8


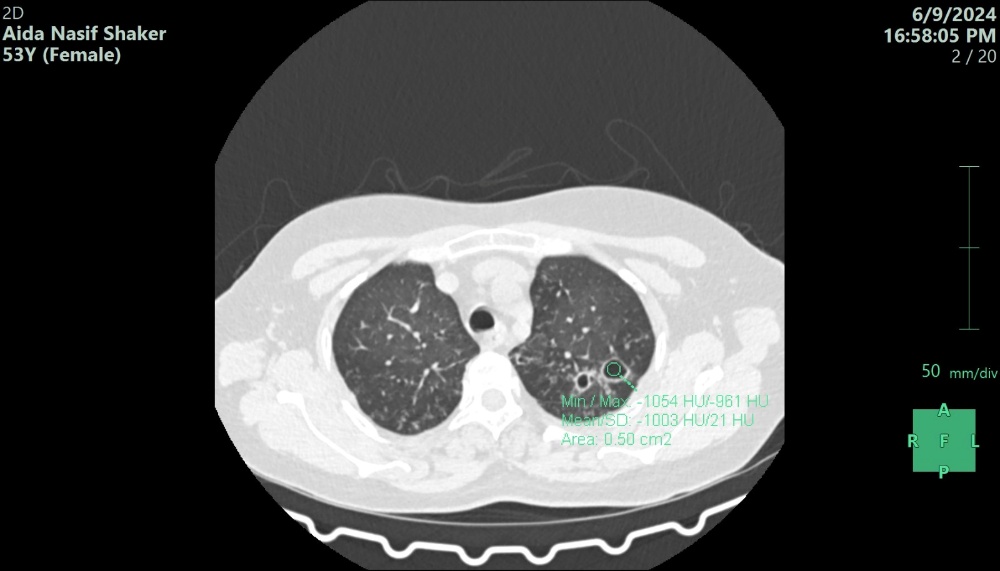


Br 48


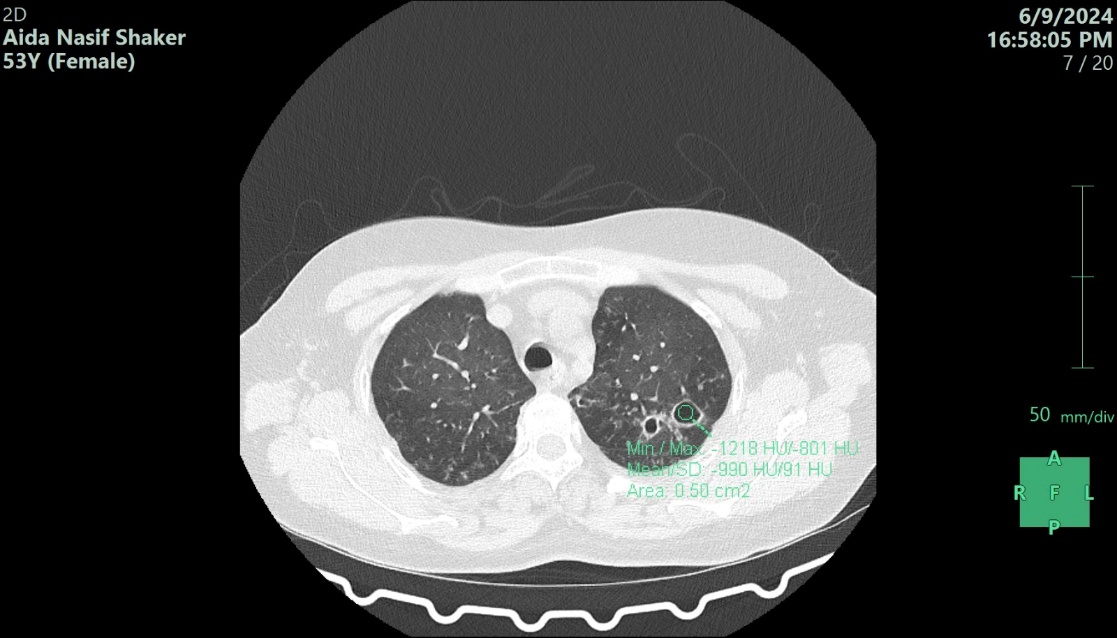


Hr 44


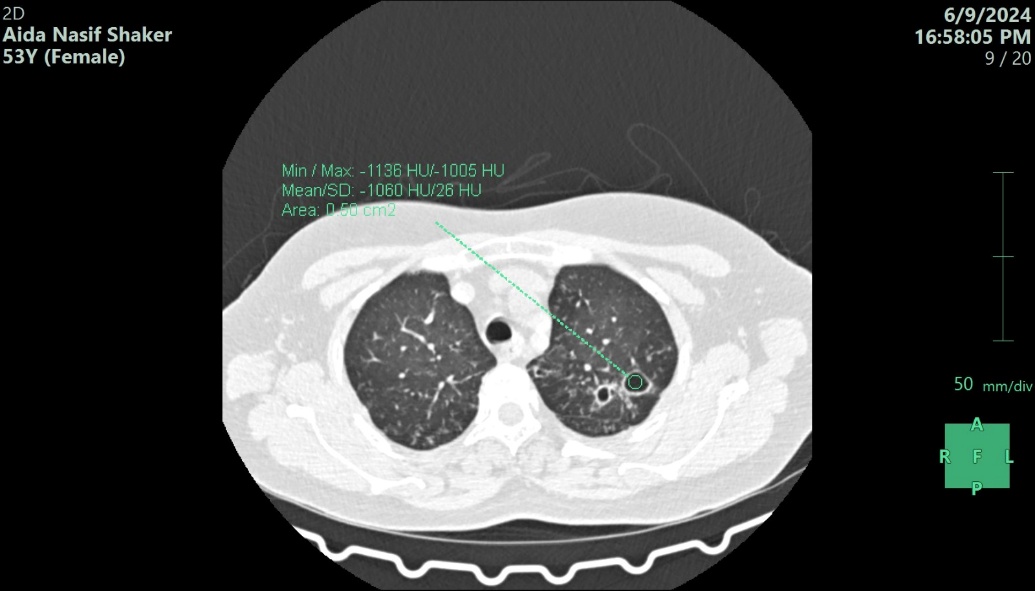


Hr 64

Supplementary Fig.9


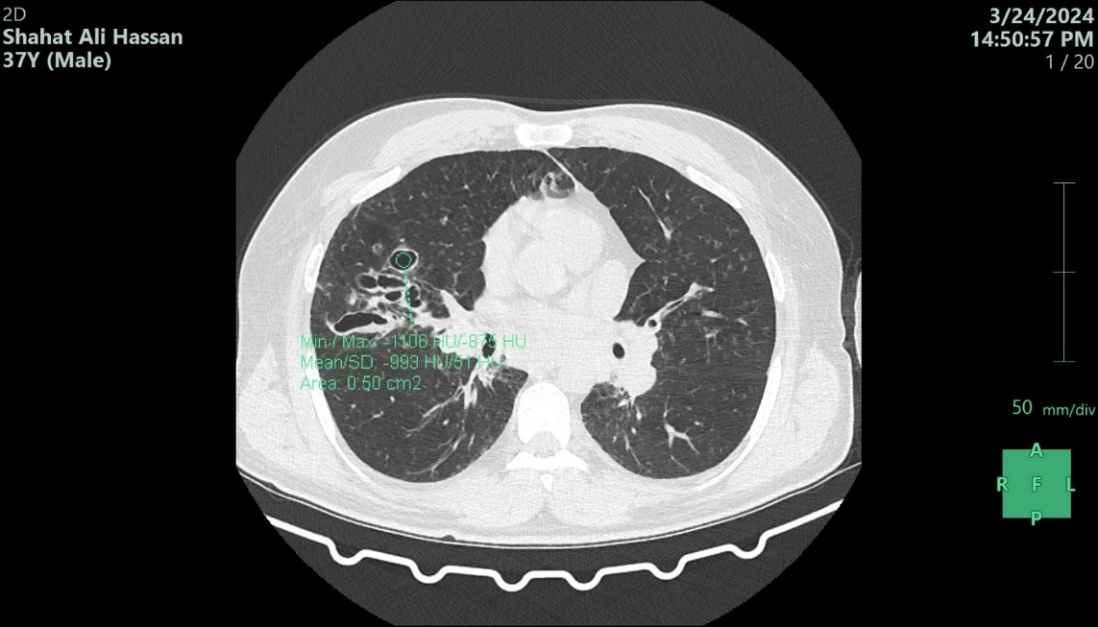


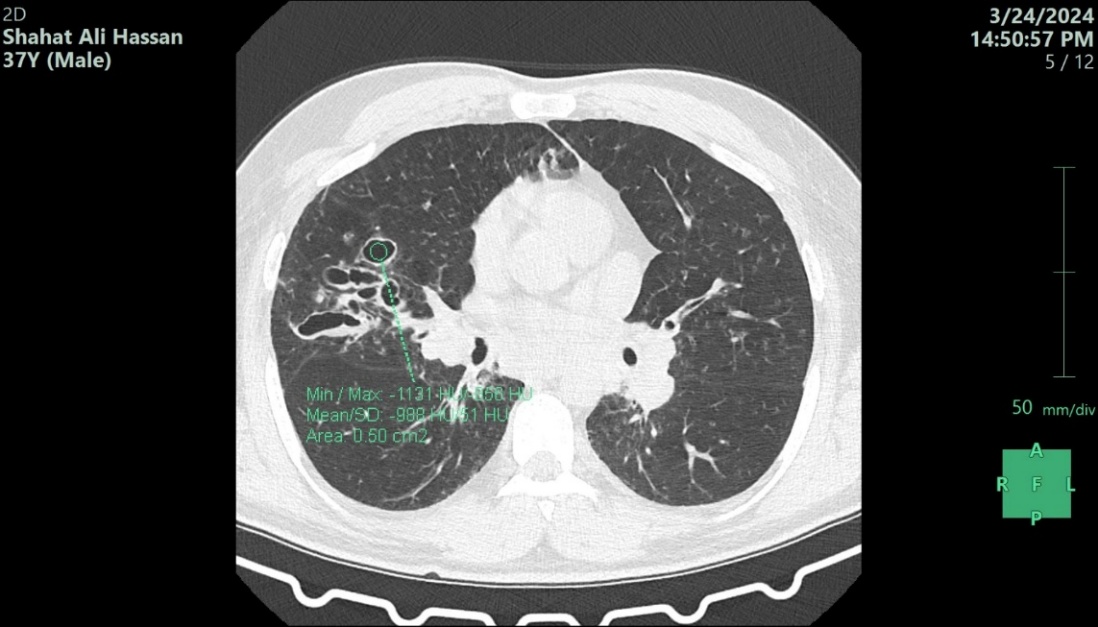


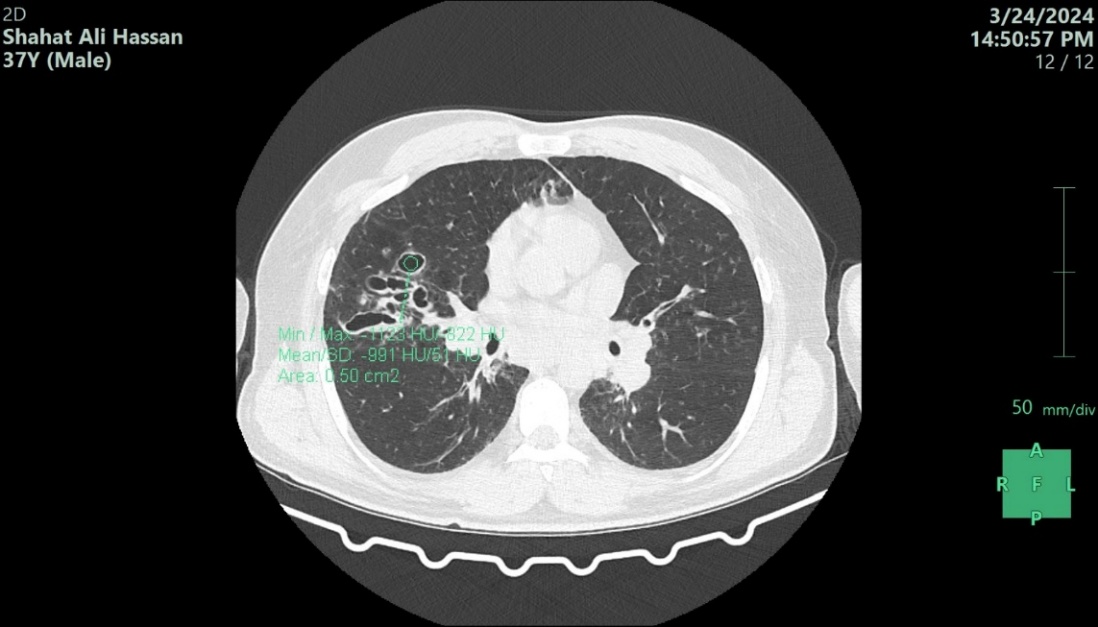


Supplementary Fig.10


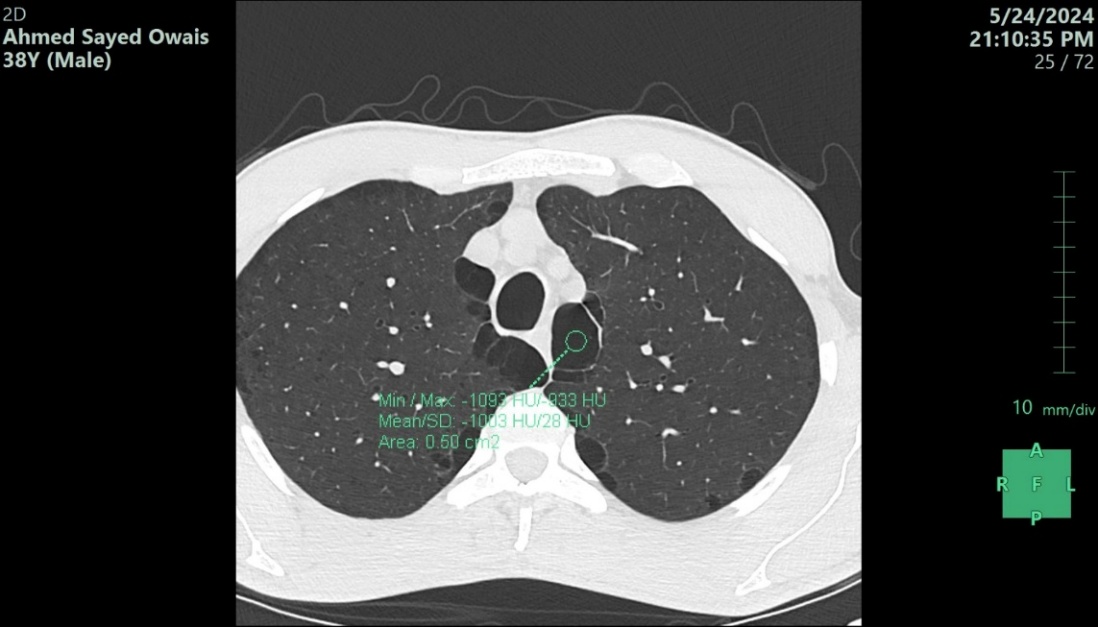


**0.25 mm**


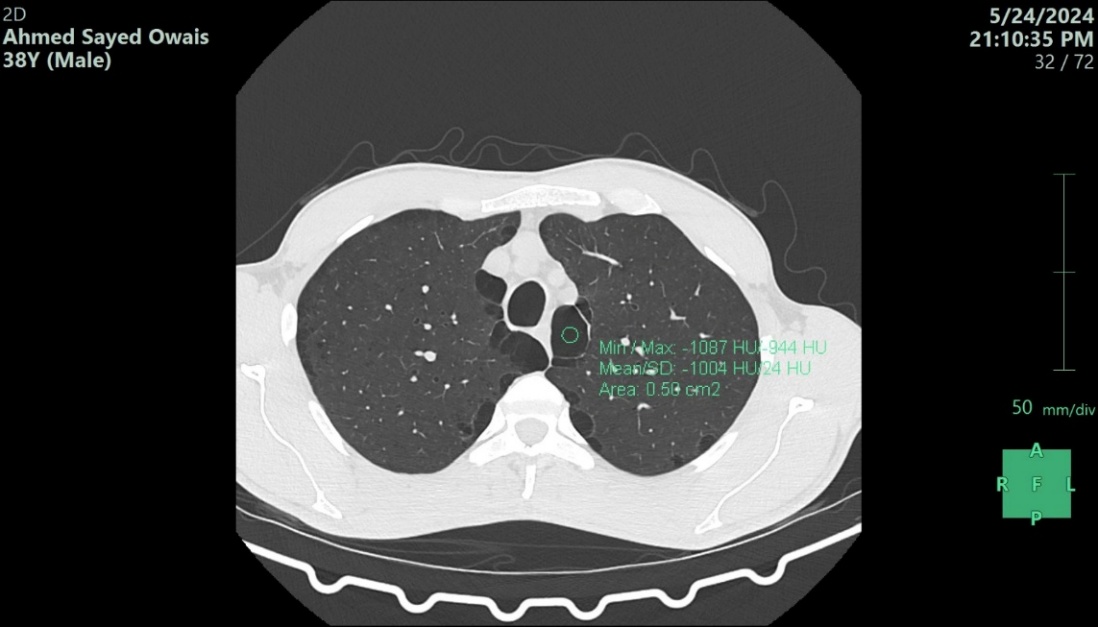


**2 mm**


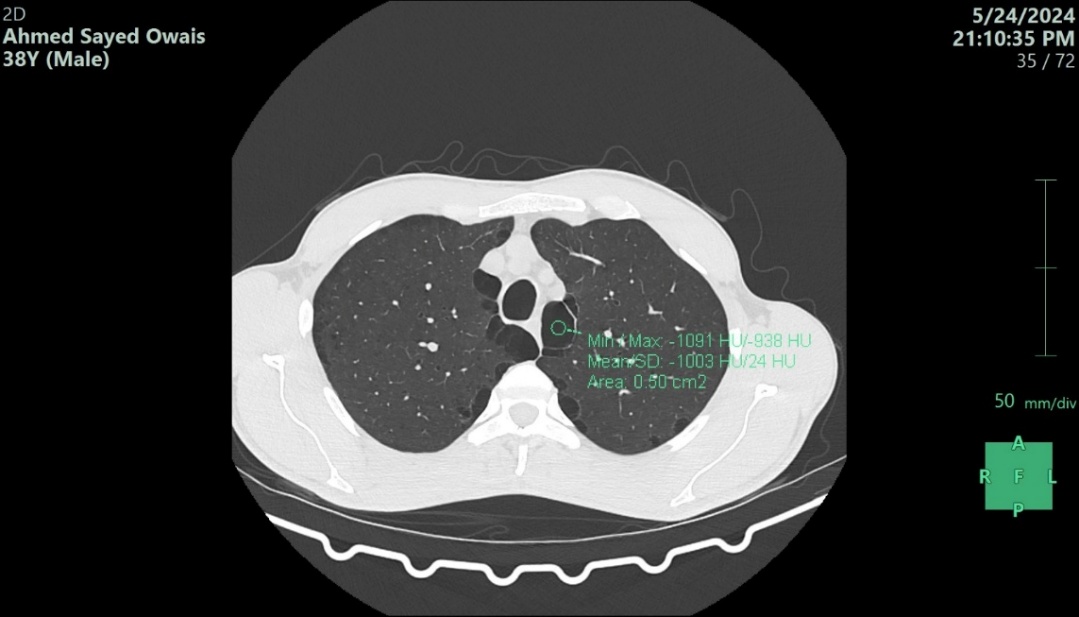


**3 mm**

Supplementary Fig.11


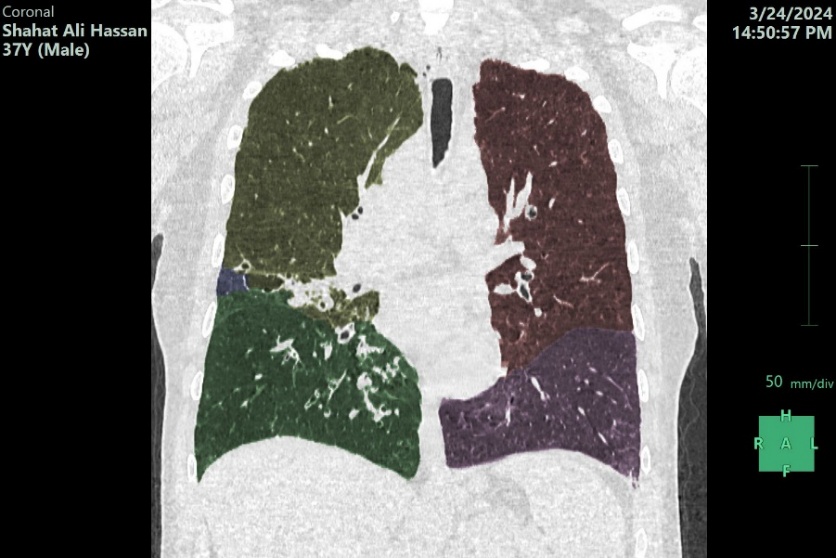

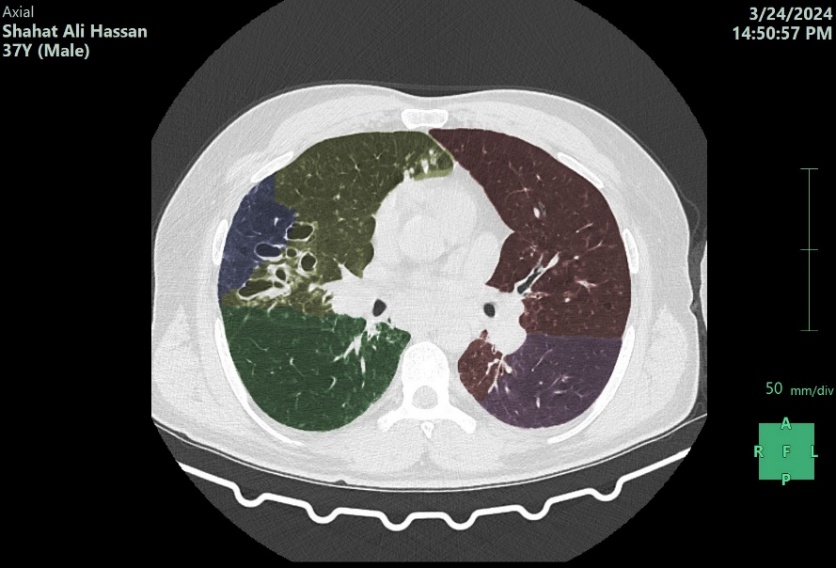


d

c

b

a


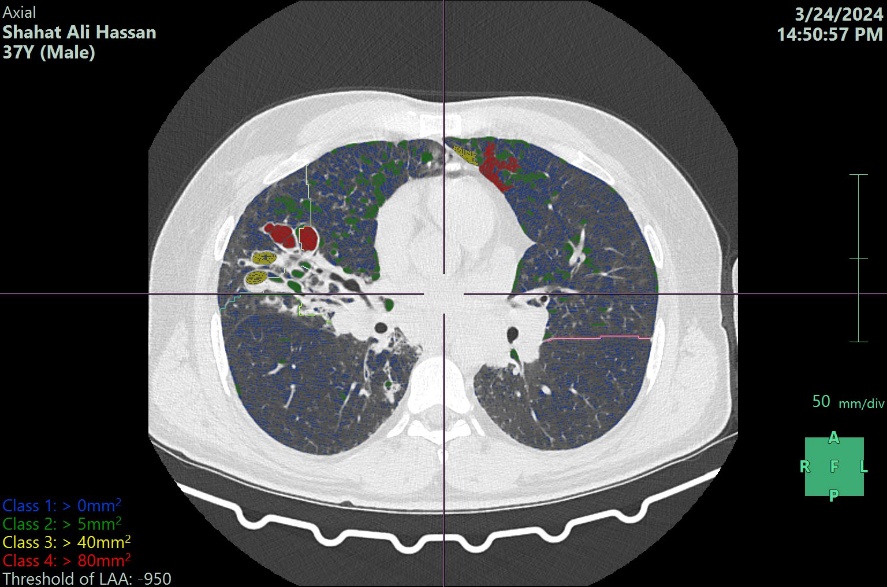

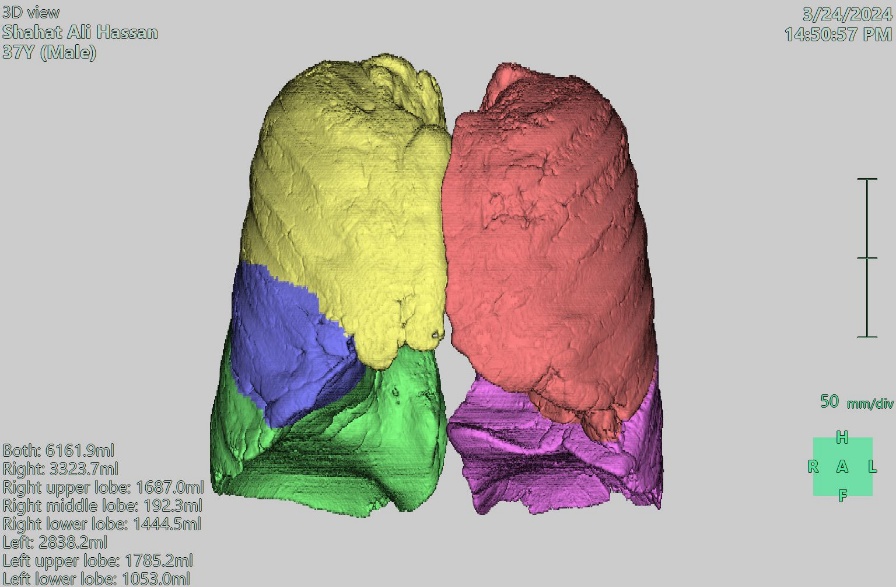


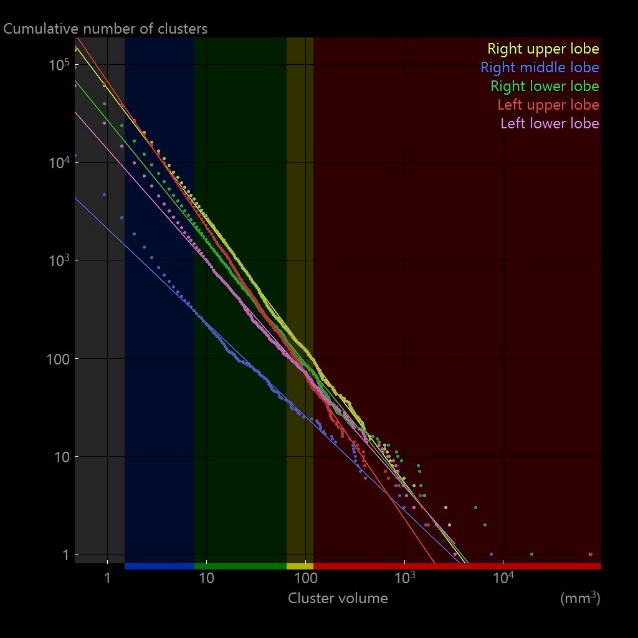

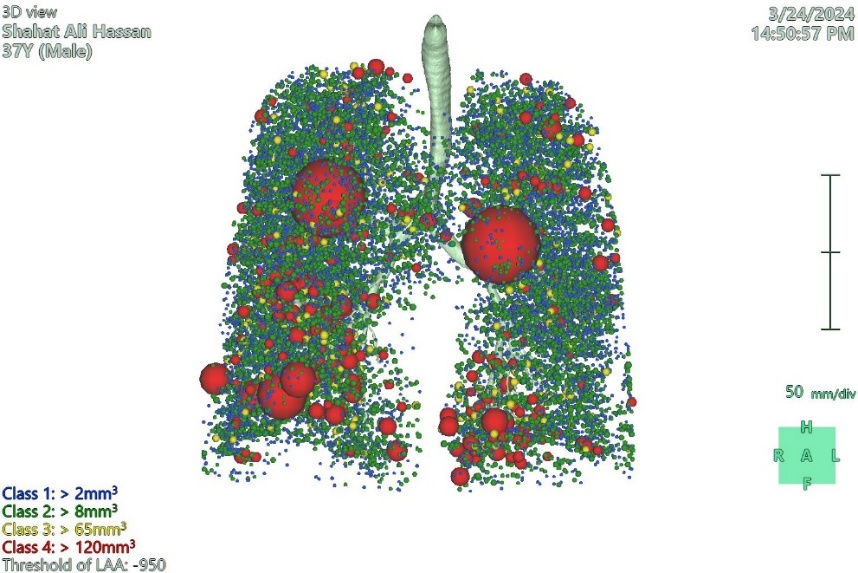


e

f

Supplementary Fig.12
